# RNA-seq analysis reveals narrow differential gene expression in MEP and MVA pathways responsible for phytochemical divergence in extreme genotypes of *Thymus daenensis* Celak

**DOI:** 10.1186/s12864-024-10164-x

**Published:** 2024-03-04

**Authors:** Hosein Ahmadi, Reza Fatahi, Zabihollah Zamani, Majid Shokrpour, Morteza Sheikh-Assadi, Peter Poczai

**Affiliations:** 1 Biotechnology and Breeding Research Group for Medicinal Plants, Department of Horticultural Science & Landscape Engineering, Faculty of Agricultural Science, University of Tehran, Karaj, Iran; 2 Finnish Museum of Natural History, University of Helsinki, Helsinki, Finland

**Keywords:** Thymol chemotype, Thymol/carvacrol chemotype, Triterpenic acids, Metabolic diversity

## Abstract

**Background:**

Here, we investigated the underlying transcriptional-level evidence behind phytochemical differences between two metabolically extreme genotypes of *Thymus daenensis.* The genotypes ‘Zagheh-11’ (thymol/carvacrol type, poor in essential oil [EO] [2.9%] but rich in triterpenic acids) and ‘Malayer-21’ (thymol type and rich in EO [3.8%]) were selected from an ongoing breeding program and then clonally propagated for further experimental use.

**Materials and methods:**

GC-MS, GC-FID, and HPLC-PDA were utilized to monitor the fluctuation of secondary metabolites at four phenological stages (vegetative, bud burst, early, and full-flowering stages). The highest phytochemical divergence was observed at early flowering stage. Both genotypes were subjected to mRNA sequencing (approximately 100 million paired reads) at the aforementioned stage. The expression patterns of four key genes involved in the biosynthesis of terpenoids were also validated using qRT-PCR.

**Results:**

Carvacrol content in ‘Zagheh-11’ (26.13%) was approximately 23 times higher than ‘Malayer-21’ (1.12%). Reciprocally, about 10% higher thymol was found in ‘Malayer-21’ (62.15%). Moreover, the concentrations of three major triterpenic acids in ‘Zagheh-11’ were approximately as twice as those found in ‘Malayer-21’. Transcriptome analysis revealed a total of 1840 unigenes that were differentially expressed, including terpene synthases, cytochrome P450, and terpenoid backbone genes. Several differentially expressed transcription factors (such as *MYB*, *bZIP*, *HB*-*HD*-*ZIP*, and *WRKY* families) were also identified. These results suggest that an active cytosolic mevalonate (MVA) pathway may be linked to higher levels of sesquiterpenes, triterpenic acids, and carvacrol in ‘Zagheh-11’. The chloroplastic pathway of methyl erythritol phosphate (MEP) may have also contributed to a higher accumulation of thymol in Malayer-21. Indeed, ‘Zagheh-11’ showed higher expression of certain genes (*HMGR*, *CYP71D180*, *β-amyrin 28-monooxygenase*, and *sesquiterpene synthases*) in the MVA pathway, while some genes in the MEP pathway (including *DXR, ispG*, and γ-*terpinene synthase*) were distinctly expressed in Malayer-21. Future efforts in metabolic engineering of MVA/MEP pathways may benefit from these findings to produce increased levels of desired secondary metabolites at commercial scale.

**Supplementary Information:**

The online version contains supplementary material available at 10.1186/s12864-024-10164-x.

## Background

The *Thymus* genus consists of more than 250 species worldwide, with eighteen species present in Iran, four of which are on the endemic list of plant species [1]. The richness of monoterpene phenols (thymol and carvacrol) in the essential oil (EO) products of thyme species makes them economically important sources for various pharmaceutical and food industries [2]. Thymol and carvacrol confer thyme species a wide range of medicinal properties, such as antimicrobial, antifungal, antidigestive, antidiabetic, and expectorant characters [2–3]. The food and drug administration (FDA) has also acknowledged the safety of these constituents as safe antioxidant agents for food stocks due to their non-noxious nature [3–4].

*Thymus daenensis* Celak. may be an ideal alternative to the commonly cultivated species (i.e., *Thymus vulgaris*), as it has a higher EO and thymol yield and better adaptation to harsh environmental conditions [5]. *T. daenensis* is also a rich repository of pentacyclic triterpenic acids (TAs), such as oleanolic acid (OA), betulinic acid (BA), and ursolic acid (UA) [6]. Some evidence suggests that TAs may be associated with anticancer, anti-inflammatory, antidiabetic, and cardioprotective properties [6–7].

Terpenoids are representatives of the most diversified and extensive classes of secondary metabolites in the plant kingdom. Terpenoids function in versatile biological roles, such as plant-pathogen interactions, osmotic regulation, and plant aroma [8]. The cytosolic mevalonate (MVA) pathway supplies precursors for biosynthesis of sesquiterpenes and triterpenes, while the 2-C-methyl-D-erythrol-4-phosphate (MEP) pathway in plastids yields primary backbones for monoterpenes and diterpenes [9]. Both pathways ultimately end up in production of isopentenyl diphosphate (IPP) and dimethylallyl pyrophosphate (DMAPP) precursors. Head-to-tail conjugation of these skeletons results in synthesis of acyclic precursors for monoterpenes (i.e., geranyl diphosphate [GPP] and farnesyl diphosphate [FPP]) [10]. Formation of basic terpenic structures is then catalyzed by multiple *terpene synthases* (*TPSs*) through oxidation and cyclization reactions in the following steps [8].

Cytochrome P450-dependent monooxygenase (*CYP450*) enzymes, encoded by the largest and oldest gene superfamilies are universally present in the vast kingdoms of biological creatures [11]. The mediation of *CYP450* enzymes in chemical reactions (e.g., oxidation, hydroxylation, isomerization, dimerization, methylation, deamination, and glycosylation) ensures that the core monoterpenes and sesquiterpenes are modified and formed in the subsequent reactions [12]. Genes belonging to the 71D and 71 A subfamilies of *CYP450* enzymes are the primary contributors to the oxidation and formation of various types of monoterpenes and sesquiterpenes [11–15]. The activity of *γ-terpinene synthase* (*TPS2*) in thyme species results in conversion of GPP into *γ*-terpinene (as the main precursor) during the initial step of synthesizing thymol and carvacrol [16]. Then, *CYP71D178*, *CYP71D179*, and *CYP71D182* continue the finalization of thymol synthesis, whereas *CYP71D180* and *CYP71D181* are proposed for carvacrol synthesis based on an examination of their substrate specificity [17].

The study of the molecular basis leading to the diversity and variation of specialized metabolites has now gained considerable attention in the field of medicinal and aromatic plant studies [18–20]. While the phytochemical compositions are largely genetically controlled, the profile of phytochemical constituents remains inherently stable, that is why we refer to them as chemotypes [21]. It is therefore necessary to study the phytochemical diversity of medicinal plants by cultivating them under the same environmental conditions [5]. Discovering the molecular drivers of chemotypes can increase the understanding on how they appear in various environmental conditions. Some of the terpene synthase genes that potentially support chemotypic variations have been characterized in *Origanum vulgare* [17] and *Thymus vulgaris* [22]. The origin of chemodiversity in plant species can be also traced back to genomic variations, such as single nucleotide or allelic polymorphisms at a single locus that encodes the enzyme responsible for producing specific chemical products. This has been suggested for the cannabinoid synthases in *Cannabis sativa* [23]. However, most reports indicate that the emergence of distinct chemotypes is typically linked to the differential expression of certain gene sets. This has been demonstrated by Padovan et al. [24] in *Eucalyptus*, Gupta et al. [19] in *Withania somnifera*, and Qiu et al. [20] in *Cinnamomum porrectum*. Numerous studies have also established that *AP2/ERF, bZIP, WRKY, MYB, NAC, MYC*, and *HD-ZIP* transcription factors can potentially regulate the promoters and expression levels of many genes involved in the formation of terpenoids and glandular trichomes in plants [25–27].

The identification of numerous enzymatic elements involved in the biosynthesis of mono-, sesqui, and tri-terpenes has greatly increased in recent years [28]. This progress is closely connected to the growing information collected from genomes and transcriptomes of different organisms [28]. In line with these advances, there is enhanced interests to improve production of terpenoids in plants or heterologous hosts, such as microbial chassis [29]. Despite the recent advances, our knowledge of transcriptomic and genomic information of *T. daenensis*, as well as key genes involved in synthesis of specialized metabolites, is still limited. The economic relevance of terpenoids has made these constituents a rich reservoir for pharmaceutical products [29]. *Thymus* species are a suitable empirical model for tracking regulatory circumstances during the expression of terpene synthases and accumulation of volatile compositions [16]. By revealing the dynamic changes of these secondary metabolites, we can also better understand the transcriptional regulators of the chemotypic variation in *T. daenensis.* The recruitment of this knowledge in targeted metabolic engineering can help us achieve the desired levels of specialized metabolites [16–17]. Indeed, transcriptome profiling integrated with metabolome analysis has opened new avenues for investigating global expression profiles, metabolic pathways, and molecular mechanisms that are likely responsible for chemical diversity [18–20, 28]. Formation of gene networks and potential regulators within these pathways can be also disentangled through different layers of ‘omics’ science [29]. The data generated by ‘Omics’ approaches is also widely recruited to develop simple sequence repeat (SSR) molecular markers for conservation, breeding, physical mapping, and QTL discovery [30]. Therefore, enriching our understanding of the genes that control the biosynthesis of volatile and non-volatile secondary metabolites in *T. daenensis* may provide a new background for molecular breeding and conservation of this species. In the present study, we performed instrumental analysis of phytochemical compounds with transcriptome profiling methods (RNA-seq and qRT-PCR) to elucidate the drivers of phytochemical divergence in two selected metabolically extreme genotypes of *T. daenensis.*

## Results

### Chemical profile of secondary metabolites

The mean shoots fresh and dry weight of ‘Malayer-21’ at early flowering stage was 142.6 g and 82 g, respectively. ‘Zagheh-11’ had a mean shoot fresh weight of 64.84 g and a shoot dry weight of 26.88 g (data not shown). The mean comparison of the main effects showed that the EO content in ‘Malayer-21’ was 1.65 times greater than that of ‘Zagheh-11’. The highest EO content in ‘Malayer-21’ and ‘Zagheh-11’ were obtained at early and full flowering stages, respectively (Fig S1). There were no noticeable differences between these two last growth stages. However, the EO content at the last two phenological stages was approximately 40% higher than that of the vegetative and bud burst stages. A mean comparison of interaction effects (genotype × phenological stage) revealed that the maximum EO content in ‘Malayer-21’ (4.5%) and ‘Zagheh-11’ (2.9%) were found at the early and full flowering stages, respectively (Fig. S1). The EO content in ‘Malayer-21’ was approximately 50% higher than that of ‘Zagheh-11’ (Fig. S1). This indicates that with progress of phenological stages, EO content gradually increases, reaches its highest content at full flowering stage, and shows significant differences with three previous growth stages (Fig. S1). However, in the genotype ‘Zagheh-11’, we observed similar trends but with two main differences. EO content reached its highest value at the early flowering stage, and at full flowering stage, a non-significant decreasing trend was found compared with early flowering stage (Fig. S1).

**Fig. 1:**
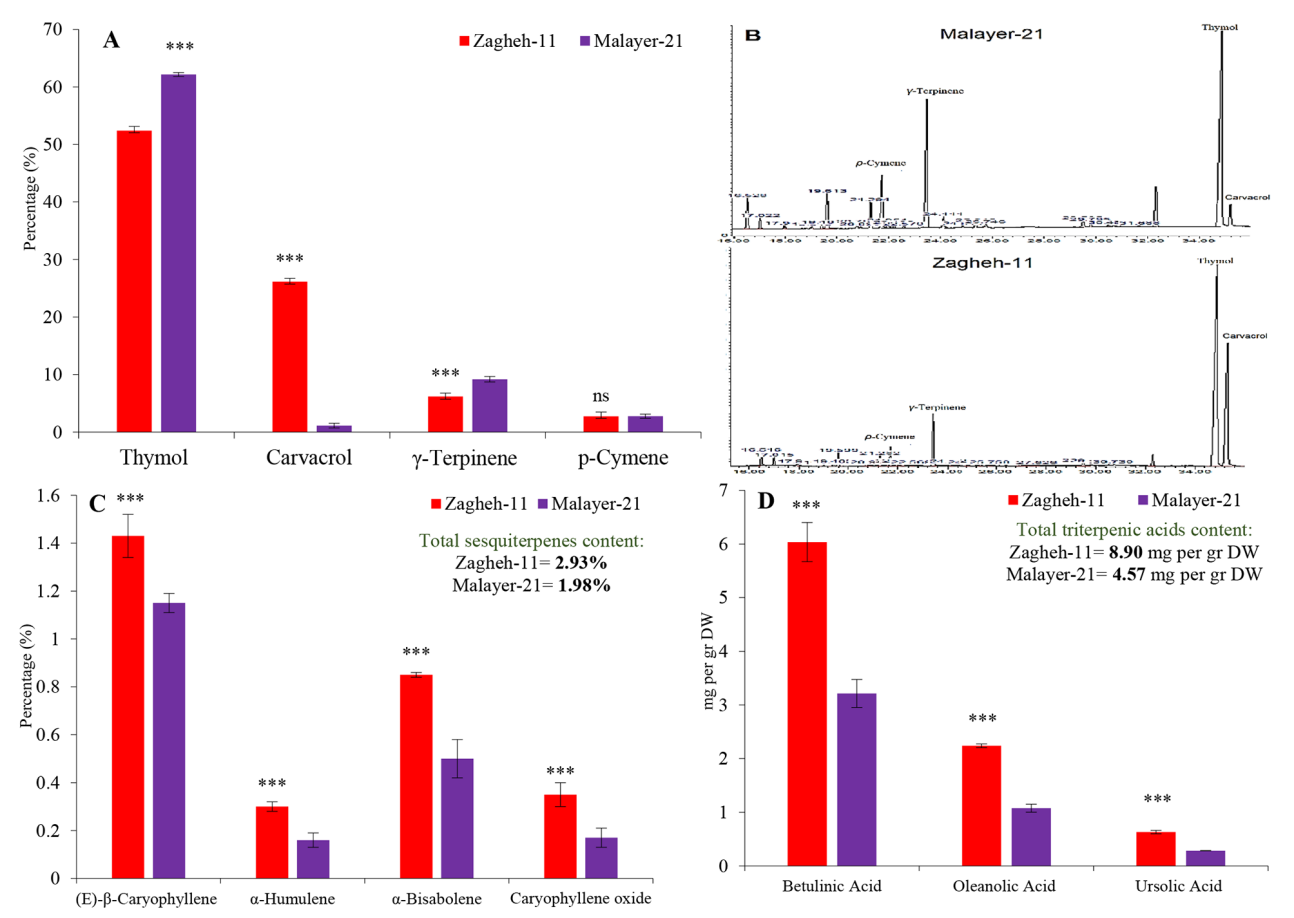
The relative percentage of main monoterpenes found in analyzed samples of essential oils (means of triplicates) (**a**), The GC-FID chromatograms of the EO of two *T. daenensis* clones (**b**), relative percentage of sesquiterpenes (**C**) and the content of major tri-terpenic acids in extreme genotypes (clones) of *T. daenensis* at early flowering stage (the best phenological stage (regarding to Table S1) (**d**). ^***^ shows significance (*p*-value < 0.001) according to independent *t*-Test

A total of 24 compounds (ranging from 95.64 to 99.59%) were characterized via GC-MS analysis of EO samples, and GC-FID was also used to determine the quantity of these constituents (Table S1; Fig. 1b). Thymol (38.98–67.98%), carvacrol (0.55–26.14%), *γ*-terpinene (8.83–21.07%), *p-*cymene (2.65–8.78%), and carvacrol methyl ether (1-5.36%) were the major components of EOs (Table S1). Overall, the content of thymol and carvacrol methyl ether were higher in ‘Malayer-21’ than ‘Zagheh-11’. However, the amounts of carvacrol, *γ*-terpinene, and *p*-cymene in ‘Malayer-21’ were lower than those of ‘Zagheh-11’. Analysis of variance (ANOVA) revealed that the main and interaction effects were statistically significant at 1% probability level for the compounds mentioned above. Means comparison of genotypes as a main effect showed that the content of carvacrol, *γ*-terpinene, and *p*-cymene in ‘Zagheh-11’ were respectively 13%, 22%, and 62% higher than those of Malayer-21. According to the means comparison of phenological stages, the highest thymol content (approximately 57%) was observed in the last three growth stages; no significant differences were observed between them. The highest carvacrol content (approximately 13.26%) was also related to early flowering stage. *γ*-terpinene (18.38%) and *p-*cymene (7.93%) showed the highest amounts during the vegetative stage. Furthermore, the *p*-cymene content of ‘Zagheh-11’ at full flowering stage was approximately 2.7 times greater than that observed for Malayer-21. Although the thymol content (52.39%) in ‘Zagheh-11’ reached its peak at the early flowering stage, there were no significant differences at full flowering stage (Table S1). In addition, ‘Malayer-21’ had the highest thymol content at the flower bud burst stage (67.98%) and exhibited significant differences from other stages (Table S1). According to the means comparison of interaction effects, we witnessed that the carvacrol content of ‘Zagheh-11’ (thymol/carvacrol chemotype) was at its highest level (26.14%) at the early flowering stage (Fig. 1b). At this point, the carvacrol content of ‘Zagheh-11’ (26.14%) was 24 times greater than that of ‘Malayer-21’ (1.12%) (Fig. 1a). ‘Malayer-21’ exhibited a relative stability for carvacrol content at different phenological stages without significant fluctuations. During the vegetative stage, *γ*-terpinene and *p*-cymene had the highest values. However, a declining trend was observed with the progress of phenological stages (Table S1).

Chromatography analysis identified four major sesquiterpenes, namely (E)-*β*-Caryophyllene, *α-*humulene, *α-*bisabolene, and caryophyllene oxide (Table S1). (E)-*β*-Caryophyllene was the predominant sesquiterpene. We found that the mean values of sesquiterpenes at the early flowering stage of ‘Zagheh-11’ were significantly higher than those of ‘Malayer-21’ according to *t*-test comparisons (Fig. 1c). Overall, the total content of sesquiterpenes in ‘Zagheh-11’ (2.93%) was approximately 48% higher than that of Maleyer-21 (1.98%) genotype. The quantity of betulinic acid (3.21–6.03 mg g^− 1^DW), oleanolic acid (1.07–2.23 mg g^− 1^DW), and ursolic acid (0.28–0.63 mg g^− 1^DW) as major triterpenic acids were also measured by HPLC-PDA at the early flowering stage. The total content and amounts of each triterpenic acid in the genotype ‘Zagheh-11’ were about 95% higher than those in ‘Malayer-21’ (Fig. 1d). Thus, triterpenic acids were more abundant in ‘Zagheh-11’.

### Functional annotation and gene ontology classification

**Fig. 2:**
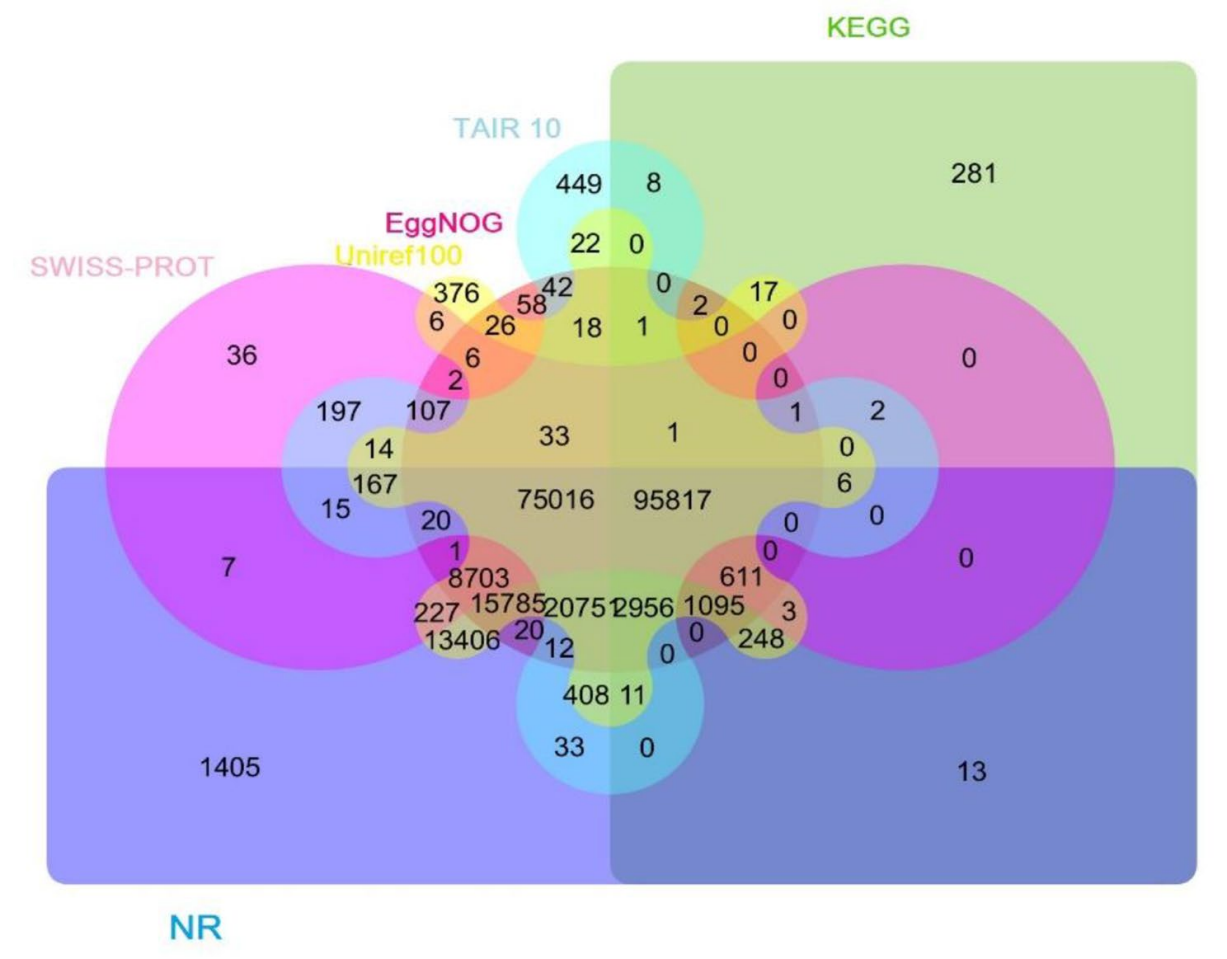
Venn diagram related to the number of BLASTx hits against six major protein databases

After trimming, a total of 97,734,816 (approximately 30 Gb nucleotide data) high-quality (Q > 30) paired-end reads were combined to be used in an optimized assembly workflow by employing EvidentialGene pipelines (see Sect. 4.5 for further details). The final assembly output file consisted of 316,786 unigenes. To annotate the reconstructed unigenes, BLASTx was performed against six major protein databases. A total of 236,736 (74.73%), 235,730 (74.41%), 221,084 (69.78%), 196,107 (61.95%), 180,998 (57.13%), and 101,074 (31.90%) unigenes from assembled transcriptome were annotated using NR, Uniref100, EggNog, TAIR10, Swissprot, and KEGG databases, respectively. According to the Venn diagram (Fig. 2), functional identification of 95,817 common unigenes was achieved by all databases mentioned above. According to gene ontology analysis, unigenes were classified into three groups (biological processes [BP], cellular components [CC], and molecular functions [MF]). The most prominent GO terms in BP category (55.82% of total unigenes) were cellular processes, metabolic processes, organic substances, primary metabolic processes, and single organism processes (Fig. S2). The number of GO terms for binding and catalytic activity in the MF category (18.17% of total unigenes) was the highest one (Fig. S2). Terms such as cells, cell parts, intracellular parts, and organelle part ranked first in the cell components (CC) category (31% of total unigenes) (Fig. S2).

### Analysis and classification of KEGG pathways

The assembled unigenes were blasted against the KEGG database. The KEGG database categorized unigenes into four major functional groups, which included metabolism, genetic information processing, cellular processes, and organismal systems groups (Fig. S3). In the metabolism group, unigenes related to global pathways (18.83% of unigenes) and the carbohydrate metabolism (4.22% of unigenes) were more abundant than other sub-categories (Fig. S3). The sub-categories such as translation (3%) and folding (3%) were prominent in the genetic information processing group (Fig. S3). In addition, signal transduction (6.07%) and environmental adaptation (2.19%) were the richest subcategories in cell processes and organismal systems groups, respectively (Fig. S3). Global and main KEGG pathways assigned for the assembled unigenes are shown in Table (S2). About 45% and 51% of the total number of identified genes in primary metabolic (25,853 unigenes) and secondary metabolic (15,234 unigenes) pathways had at least one orthologue (Table S2).

A total of 2,096 unigenes were implicated in the metabolism of terpenoids and polyketides. The data revealed that each enzyme involved in these pathways could be encoded by multiple unigenes (gene isoforms). The most complete metabolic maps in the secondary metabolites category (Table S3) were flavonoids (possessing 74% of identified genes) and terpenoids backbone (possessing 58% of identified genes). As shown in Table S2 and S3, 776 assembled unigenes (orthologues) were assigned to 31 (out of 53) genes that were already identified in the reference pathway of terpenoid backbones. Almost all genes associated with subsequent steps of synthesizing isoprenoids through MVA and MEP pathways were identified in the *T. daenensis* transcriptome. Table (S4) provides information on the names, KO ID, and EC number of these genes. It should be noted that 6 out of 23 genes (26%) and 11 out of 66 genes (17%) were also mapped to monoterpenoid and sesquiterpenoid/triterpenoid pathways, respectively (Tables S3 and S4).

### Identification and expression profiling of DE unigenes (RNA-seq and qRT-PCR data)

**Fig. 3:**
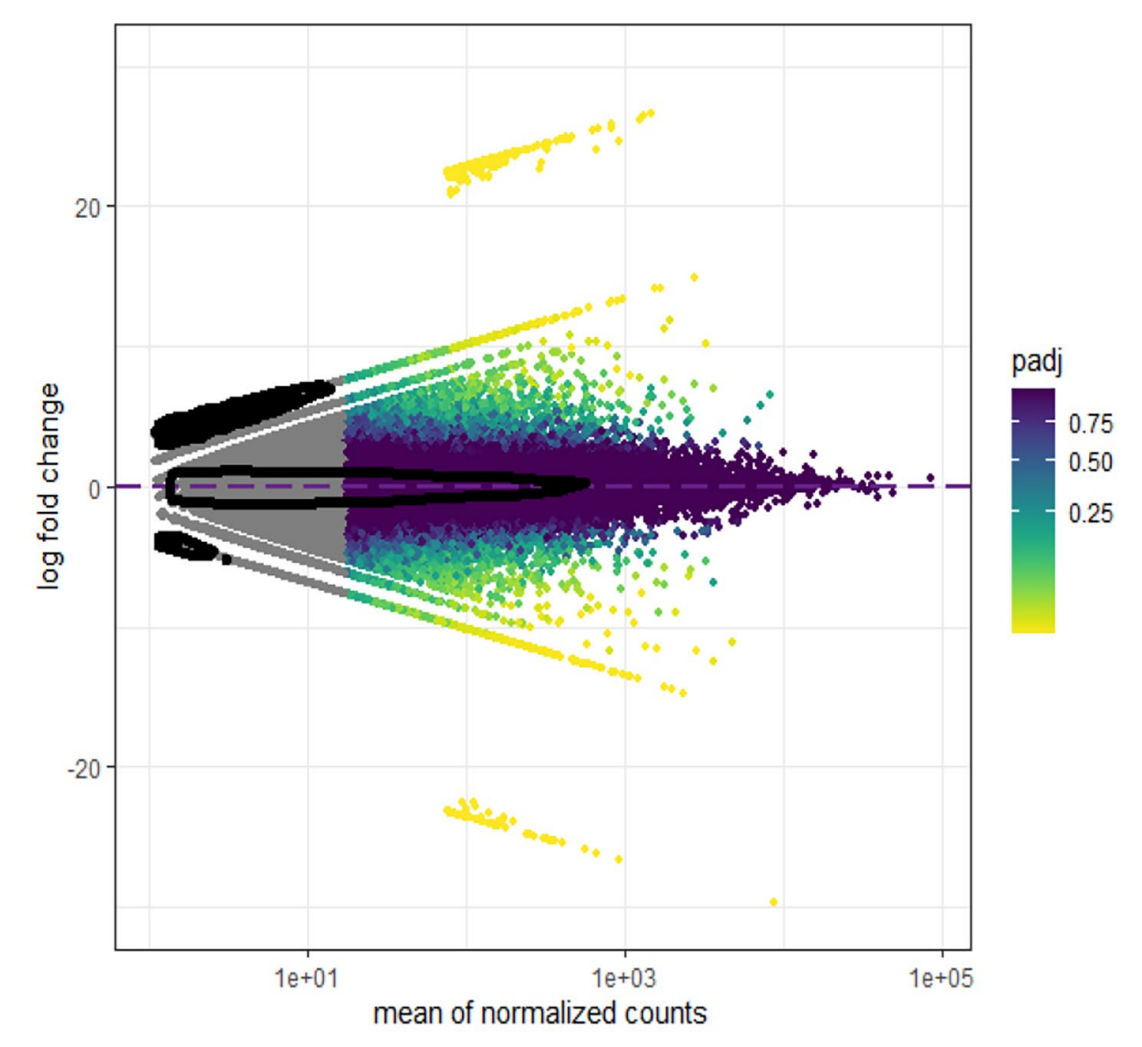
MA plot of normalized counts of differentially expressed unigenes. A total of 1840 unigenes were identified as DE unigenes with log_2_FC greater than 2 and FDR < 0.05

**Fig. 4:**
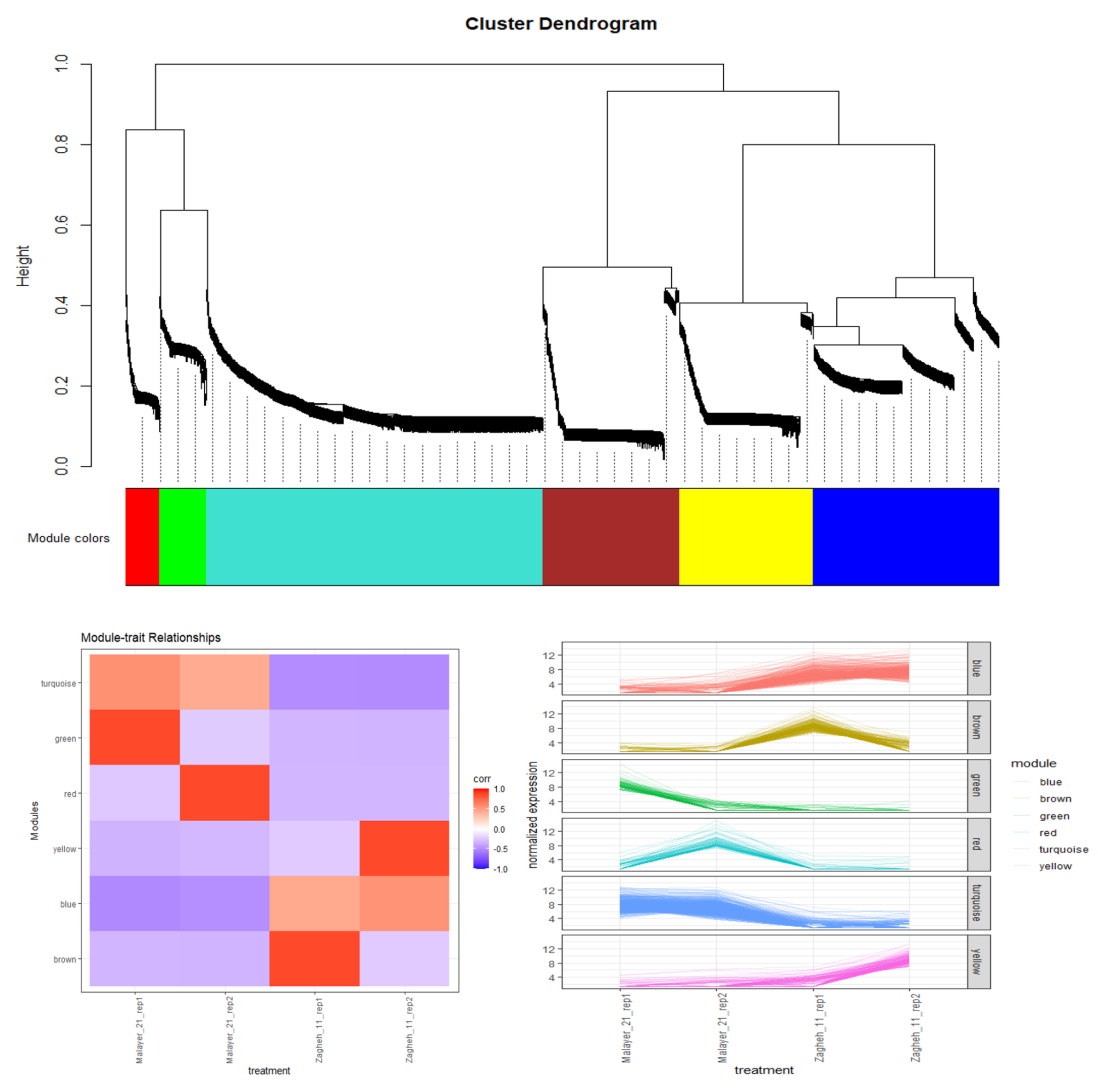
Weighted genes co-expression network, correlation of modules and treatments, and normalized expression values of unigenes related to each module

**Fig. 5:**
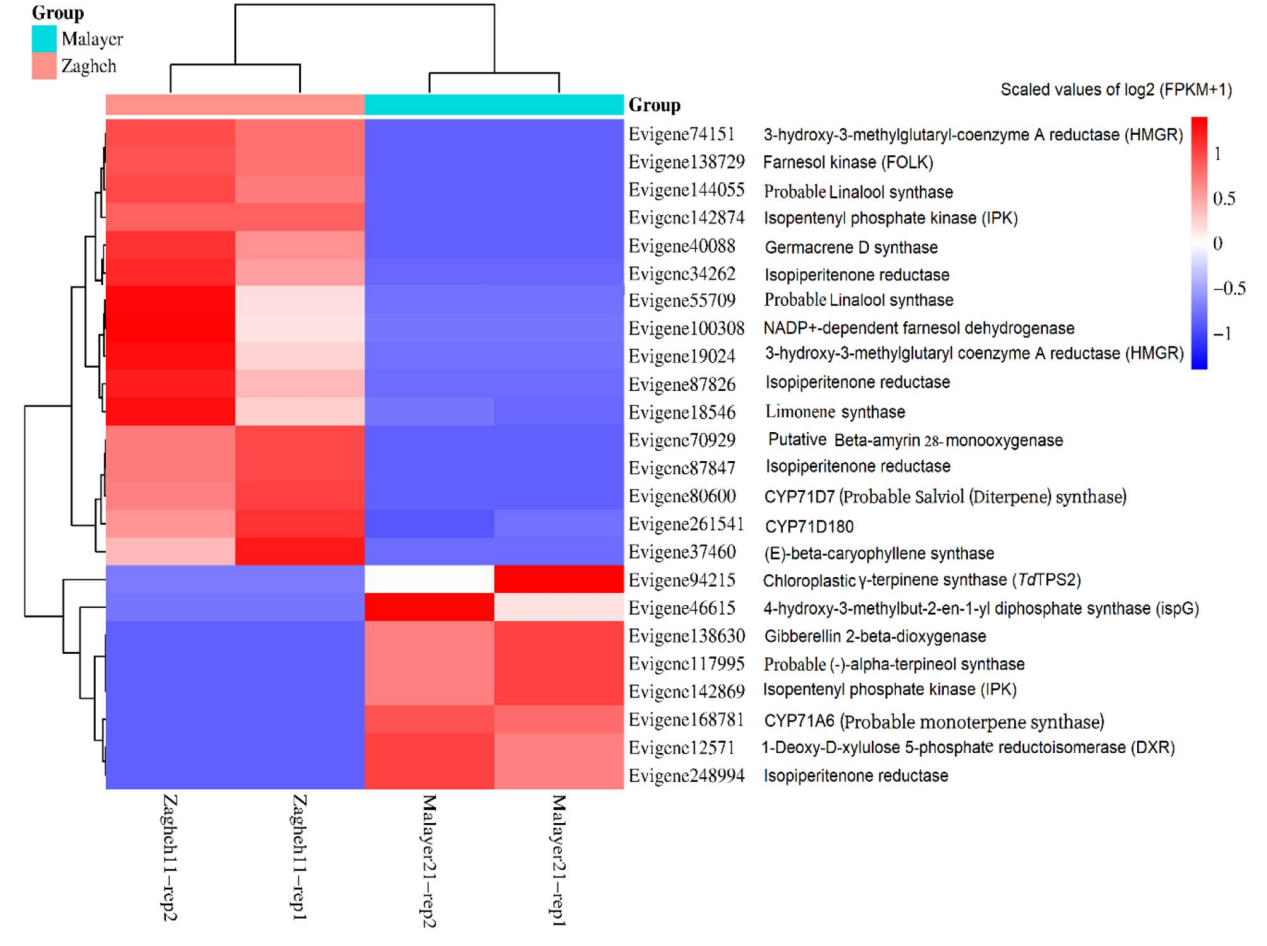
Heatmap plot showing the normalized values of log_2_ (FPKM + 1) related to differentially expressed unigenes which were involved in terpenoids biosynthesis pathways in two chemotypes of *T. daenensis*

**Fig. 6:**
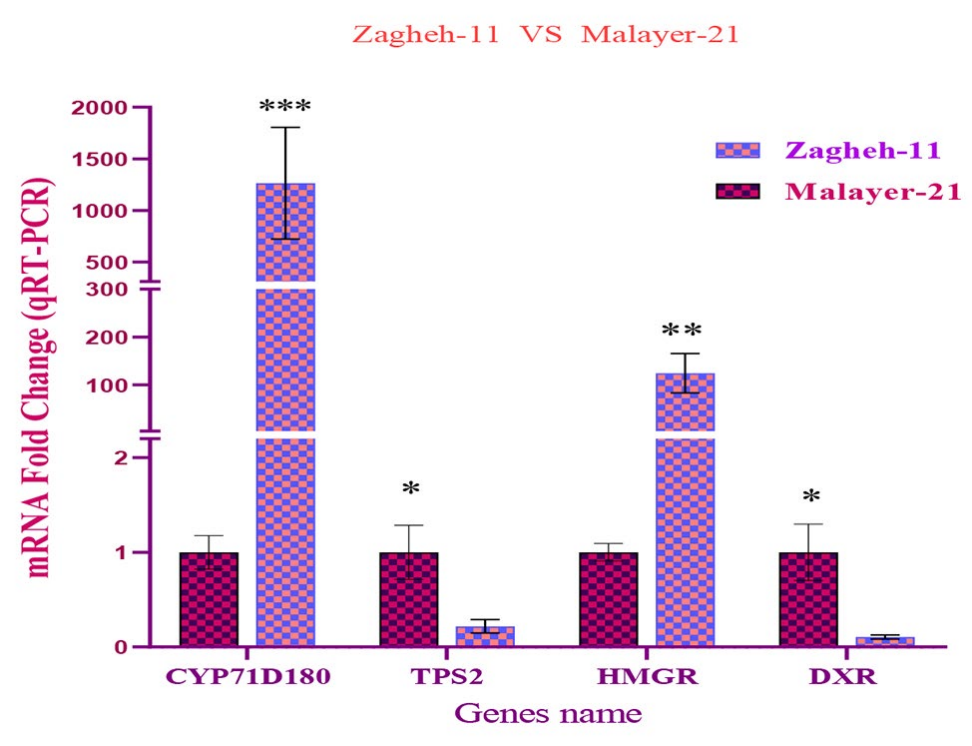
mRNA fold change values (Zagheh-11 vs. Malayer-21 (reference group)) of four key unigenes involved in metabolic divergence (terpenoids biosynthesis) of *T. daenensis* extreme genotypes obtained by qRT-PCR analysis. ^***^, ^**^, ^*^ signs denote significant differences at 0.001, 0.01, and 0.05 levels according to *t*-Test pairwise mean comparisons

Out of 316,786 assembled unigenes, 1840 unigenes had differential expression patterns among the studied samples. The MA diagram (Fig. 3) display the distribution and mean normalized counts of total assembled unigenes and DE unigenes, respectively. Differential expression analysis revealed that 948 unigenes were upregulated and 892 unigenes were downregulated in ‘Zagheh-11’ compared with ‘Malayer-21’ (Fig. 3). Fig. S4 shows clustering results that correspond to normalized expression values (− 1.5 to 1.5) of DE unigenes (FDR < 0.05). In the genotypes studied, six distinct gene expression patterns (clusters) were illustrated. Unigenes contents of clusters 2 (707 unigenes), 5 (109 unigenes), and 6 (95 unigenes) showed overexpression patterns in high EO containing/thymol type genotype (Malayer-21) (Fig. S4). On the other hand, unigenes presented in cluster 1 (406 unigenes), 3 (300 unigenes), and 4 (285 unigenes) were abundantly expressed in ‘Zagheh-11’ (Fig. S4). Similarly, weighted co-expression network (WCGNA) analysis using topological overlap matrix (TOM) segregated unigenes into six merged modules. This clustering was well-consistent with the above-mentioned non-weighted clustering method (Fig. 4). Blue (400 unigenes), brown (294 unigenes), and yellow (294 unigenes) modules showed high correlations and overexpression patterns in ‘Zagheh-11’, while turquoise (724 unigenes), red (74 unigenes), and green (101 unigenes) modules were correlated with ‘Malayer-21’ genotype (Fig. 4). The blue, brown, green, and turquoise modules had more association with the chemical profile of these genotypes. The information generated by multiple annotation database was scrutinized to identify probable functional roles of DE unigenes in biosynthesis pathways of secondary metabolites. A total of 24 out of 1840 DE unigenes were identified to be involved in the synthesis of terpenoids (Fig. 5). A heatmap plot revealed that 8 and 16 unigenes were overexpressed in ‘Malayer-21’ and ‘Zagheh-11’, respectively (Fig. 5). The ‘Malayer-21’ genotype showed an extremely high expression values for some unigenes related to *DXR* (Evigene12571, log_2_FC = 9.59), *ispG* (Evigene46615, log_2_FC = 25.14), and *γ-terpinene synthase* (Evigene94215, log_2_FC = 23.48) enzymes when compared with ‘Zagheh-11’ (Fig. 5). Overall, expression levels of monoterpene synthases, such as *linalool synthase*, *limonene synthase*, and *isopiperitenone reductase* were higher in ‘Zagheh-11’. According to RNA-seq data, ‘Malayer-21’ had approximately 1,468 times greater expression values for *gibberellin 2-beta-dioxygenase* (Evigene138630) (Fig. 5). We found that some important unigenes related to biosynthesis of sesquiterpenes and triterpenes were highly overexpressed in ‘Zagheh-11’. *Beta-amyrin 28-monooxygenase* (log_2_FC = 23.12), *NADP*^*+*^*-dependent farnesol dehydrogenase* (log_2_FC = 9.44), *farnesol kinase* (log_2_FC = 9.66), *germacrene* D *synthase* (log_2_FC = 8.72), *caryophyllene oxide synthase* (log_2_FC = 8.94), and 3-hydroxy-3-methyl-glutaryl-coenzyme A reductase (two unigenes, log_2_FC = 7.8) were over-expressed in ‘Zagheh-11’. In the genotype ‘Zagheh-11’, the unigene Evigene261541 (*CYP71D180*) had an expression value that was 630 times greater than Malayer-21. The unigenes mentioned above were statistically significant at FDR < 0.01 level. To validate the RNA-seq experiment, real-time PCR expression patterns of four key genes were also monitored using isoform-specific primers in the studied genotypes. The gene expression patterns in qRT-PCR were similar to those generated by RNA-seq data analysis. In pairwise comparison of qRT-PCR results, we observed that in ‘Zagheh-11’ genotype, *CYP71D180* and *HMGR* genes had 1,264 (*p*-value < 0.001) and 124 (*p*-value < 0.01) times greater mRNA expression values (Fig. 6), respectively. According to unpaired *t*-test comparison (‘Malayer-21’ vs. ‘Zagheh-11’), real-time PCR analysis could also reveal a 5- and 10-fold increase in gene expression levels of *Td*TPS2 (*p*-value < 0.05) and *DXR* (*p*-value < 0.05), respectively (Fig. 6).

### GO enrichment analysis of differentially expressed genes

**Fig. 7:**
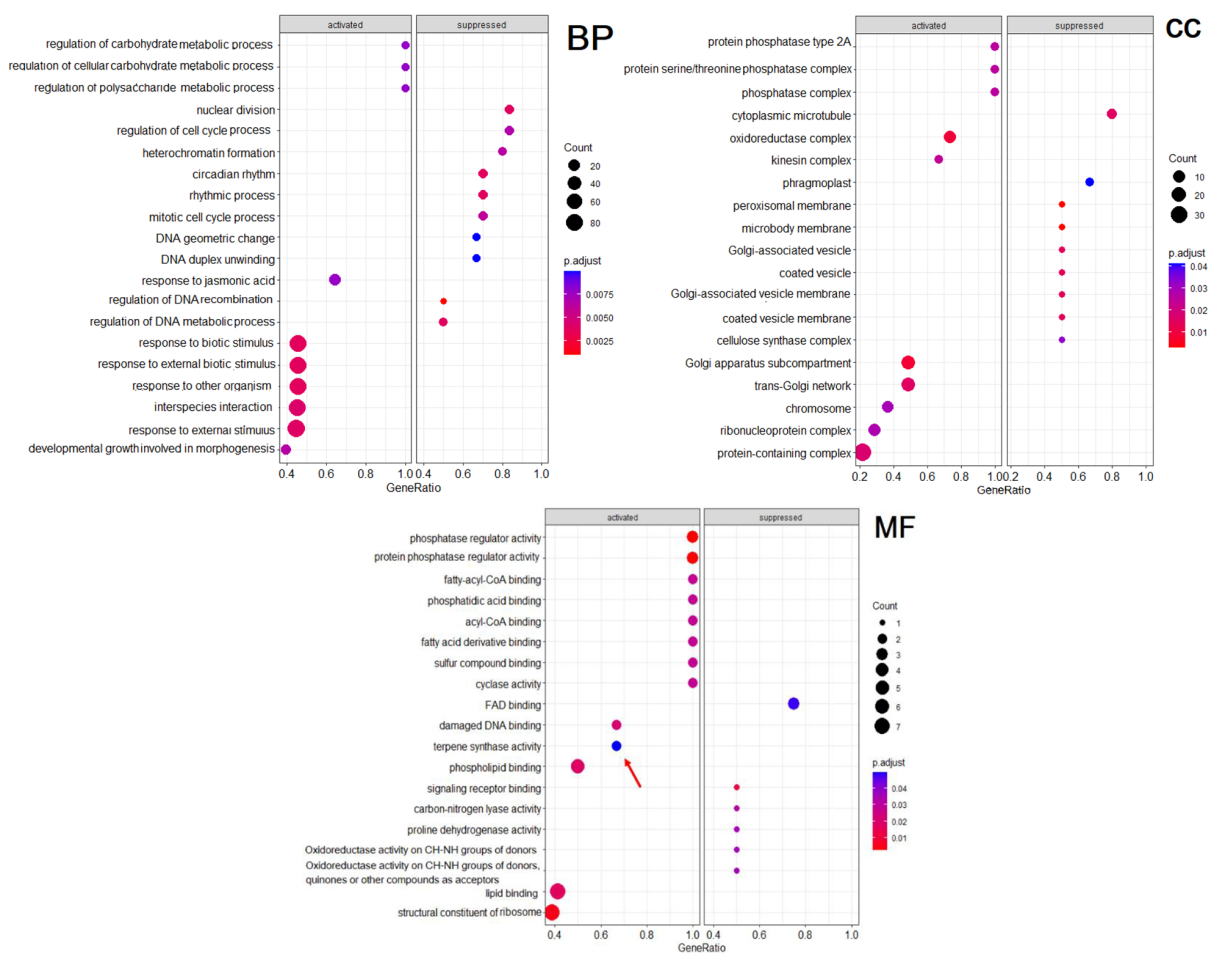
Parametric analysis of gene set enrichment related to differentially expressed (DE) unigenes in three main GO categories (BP, MF and CC) in Zagheh-11 vs. Malayer-21 pairwise comparisons. Only 20 top GO terms with *p*-adjusted < 0.05 are presented

Parametric gene ontology enrichment analysis was conducted by using log_2_FC values of DE unigenes. The top 20 GO terms (activated or suppressed) with *p-adjusted* < 0.05 are shown in Fig. 7. In ‘Zagheh-11’ vs. ‘Malayer-21’ pairwise comparisons, some GO terms such as responses to biotic and abiotic stimuli, response to other organisms, response to jasmonic acid, response to external stimulus, carbohydrate metabolism, and interspecies interactions in biological process (BP) category seemed to be activated. On the other hand, terms related to DNA regulation and metabolism were suppressed in ‘Zagheh-11’ (Fig. 7). Within the molecular function (MF) category, the most enriched GO terms in ‘Zagheh-11’ were terpene synthase activity, phosphate regulator activity, fatty acids binding, and sulfur binding. Signal receptor binding was the most significant GO term suppressed in ‘Zagheh-11’ (Fig. 7). Oxidoreductase complex, golgi apparatus sub-compartment, and trans-golgi network were also the most enriched cellular functions in the cellular component (CC) category (Fig. 7). Using the quantity information of unigenes, over-representation gene set enrichment analyses was performed based on gene ontology. The results are shown in bubble plots (Fig. S5). The most important and over-represented GO terms in BP category were metabolic process, biological process, responses to stimulus, photosynthesis, and isoprenoids metabolic processes (Fig. S5). In the MF category, there were significant differences between genotypes in GO terms such as ATP-binding, carbohydrate binding, and catalytic activity (Fig. S5). The interaction of GO terms involved in biological processes are presented in Fig. S6.

### Enrichment analysis of KEGG pathways

By enriching KEGG pathways, we identified metabolic pathways that were over-represented and included significantly overexpressed unigenes. In pairwise comparison of ‘Zagheh-11’ vs. ‘Malayer-21’, 23 KEGG pathways were recognized as significantly enriched routes (at FDR < 0.05) (Table 1). There were significant differences between genotypes in primary (e.g., carbon fixation, photosynthesis, carbohydrates, fatty acids, sulfur, and amino acid metabolism) and secondary (e.g., terpenoids, carotenoids, flavonoids, alkaloids, and stilbenoids) metabolic pathways (Table 1). The top 5 over-represented pathways were metabolic pathways, biosynthesis of secondary metabolites, terpenoids backbone biosynthesis, monoterpenoid biosynthesis, flavonoid biosynthesis, and carbon fixation in photosynthetic organs (Table 1). Most of the background gene contents in metabolic maps were covered by unigene orthologs, as seen in pathways such as monoterpenoid biosynthesis (100%), carbon fixation in photosynthetic organs (57%), stilbenoid/gingerol biosynthesis (43%), flavonoid biosynthesis (41%), and photosynthesis (40%). Indeed, these pathways received the highest enrichment scores (Table 1).

**Table 1:** KEGG pathways over-representation enrichment analysis of differentially expressed genes in pairwise comparison of Zagheh-11 vs. Malayer-21 using KOBAS online web server (http://kobas.cbi.pku.edu.cn)

KEGG Maps	Number of background gene content	Number of reference genes covered by DE unigenes (orthologs)	Enrichment score	FDR value
Metabolic pathways	2246	281	0.13	1.33E-42
Biosynthesis of secondary metabolites	1107	149	0.13	1.71E-24
Terpenoid backbone biosynthesis	60	11	0.18	0.002
Monoterpenoid biosynthesis	8	8	1	1.82E-06
Flavonoid biosynthesis	22	9	0.41	5.33E-05
Carbon fixation in photosynthetic organs	69	39	0.57	3.40E-23
Carbon metabolism	273	67	0.25	1.32E-22
Photosynthesis	77	31	0.4	2.35E-15
Glyoxylate and dicarboxylate metabolism	78	29	0.37	1.00E-13
Glycolysis / Gluconeogenesis	116	26	0.22	2.37E-08
Biosynthesis of amino acids	251	37	0.15	2.62E-07
Plant-pathogen interaction	170	29	0.17	4.92E-07
Pentose phosphate pathway	58	16	0.28	1.90E-06
Fatty acid biosynthesis	43	11	0.26	0.0002
Cysteine and methionine metabolism	121	17	0.14	0.001
Isoquinoline alkaloid biosynthesis	22	7	0.32	0.001
Fatty acid metabolism	69	12	0.17	0.001
Phenylalanine metabolism	32	8	0.25	0.002
Fructose and mannose metabolism	64	11	0.17	0.0029
Glutathione metabolism	102	12	0.12	0.021
Stilbenoid, and gingerol biosynthesis	7	3	0.43	0.02
Carotenoid biosynthesis	29	5	0.17	0.05
Sulfur metabolism	42	6	0.14	0.05

### Transcription factors and expression patterns

**Fig. 8:**
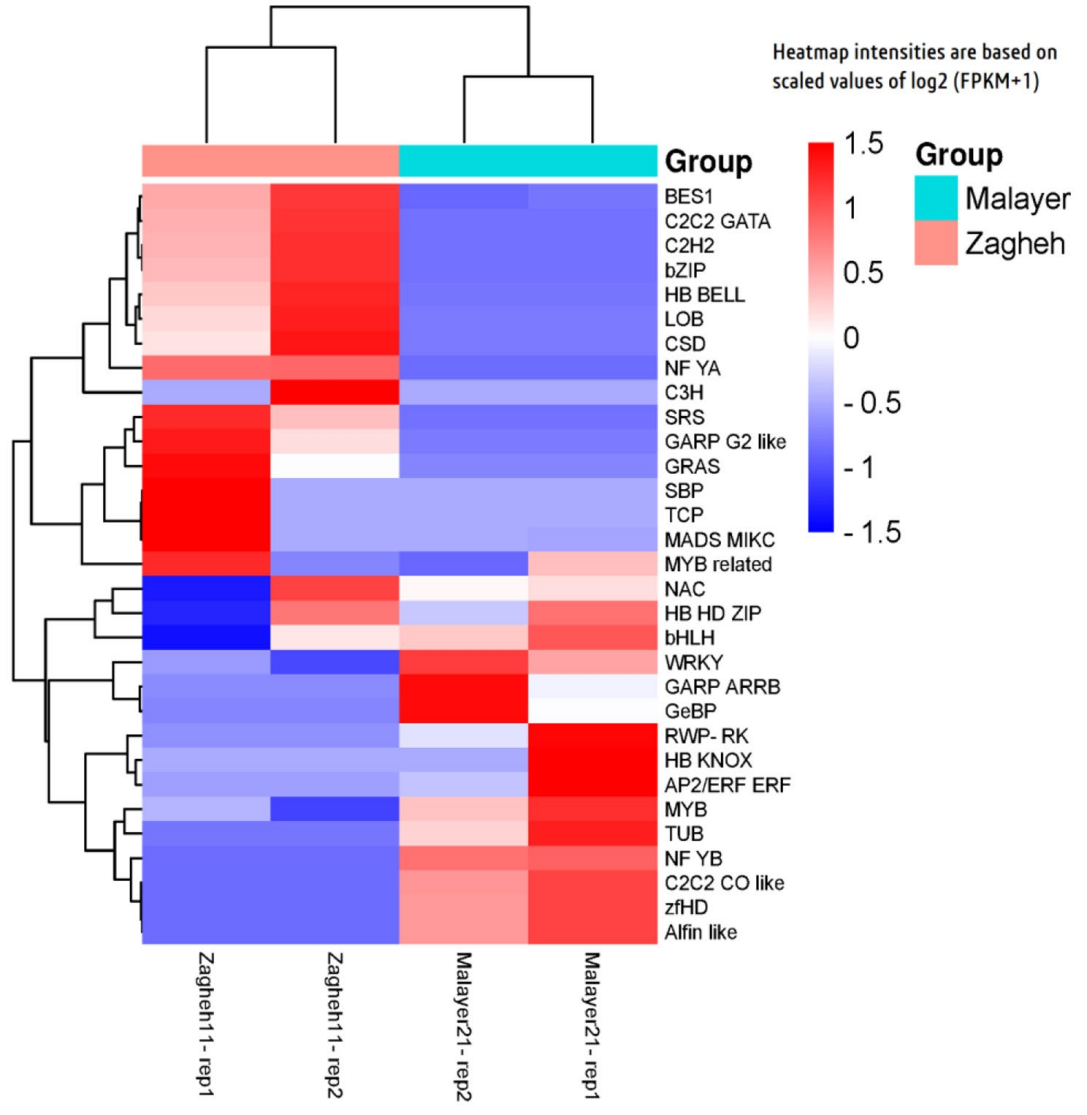
Heatmap plot showing expression patterns of differentially expressed TFs families. Note that mean expression values of unigenes related to each TF family was used to generate heatmap plot

A BLASTx search for known transcription factors in 165 species revealed 9066 unigenes as putative TFs that were apportioned in 61 families with high homology levels (amino acid identity > 80%) (Fig. S7). The most abundantly expressed TF families were *AP2/ERF* (6.42%), *C2H2* (6.42%), *bHLH* (6.22%), *WRKY* (5.84%), *MYB* (5.81%), *MYB*-related (5.79%), *C3H* (4.92%), *NAC* (4.72%), *B3* (3.82%), and *bZIP* (3.38%) (Fig. S7). The studied samples showed differential expression patterns for 50 unigenes belonging to 31 TF families. Average calculations were performed to determine the overall expression value of each transcription factor families (those families with more than one unigene). The expression patterns of all DE unigenes are presented in Fig. (8). Some influential transcription factors (*AP2*/*ERF*, *bHLH*, *WRKY*, *MYB*, *MYB*-related, *HB*-*HD*-*ZIP*, and *NAC*) in regulation of secondary metabolites and secretory glands biogenesis were over-expressed (FDR < 0.05) in samples related to Malayer-21, except for the *bZIP* transcription factor (Fig. 8).

## Discussion

In our recently published paper, we developed an efficient workflow for *de novo* assembly of transcriptomic data, which is also used here for DE analysis. The nucleotide data were assembled by utilizing five the state-of-art assembly tools, outputs combined, clustered through CD-HIT, and finalized by EvidentialGene pipelines [31]. In this ensemble workflow, EvidentialGene won the competition among seven assemblers based on the normalized scores of 16 evaluation metrics [31]. The superiority of EvidentialGene is due to the fact that this software monitored the deduced amino-acid sequences, concurrently examined the quality of coding regions, UTRs, gaps, and also checked for the presence of start-stop codons [31]. This superiority also appeared in the annotation results, in which over 74% of unigenes were functionally identified, surpassing the reported ratios from other studies (Fig. 2) [31]. Based on the reference KEGG maps, a large portion of the unigenes (15,234) were attributed to secondary metabolic processes. The significance of pathway analysis in elucidating the biosynthetic processes driving formation of drug components was highlighted by the large number of unigenes identified through the KEGG database [32]. In EO-bearing plants, screening biosynthetic pathways of terpenoids, which is poorly understood due to the lack of complete genomes, is a key focus of studies [28]. In the present work, most of the enzymatic elements directing the biosynthetic steps of terpenoids backbones (a total of 776 unigenes) had more than one unigene (Table S3). The identification of paralogues genes encoding different enzymatic isoforms that catalyze subsequent chemical reactions was facilitated by these outcomes. These findings augment the possibility that these unigenes may undergo various types of duplication events. Gene-duplication events typically originate from molecular circumstances, such as whole genome or local (tandem) duplications [33]. Gene duplications have critical roles in expanding the diversity of specialized metabolites in plant species [33]. There are two potential impacts that may be caused by these events, namely functional divergence of the enzymatic elements due to nonsynonymous mutations which usually occur during relaxed selection pressures and changes in gene dosage (copy number variations [CNVs]) [34]. For example, an experimental investigation unraveled that gene copy numbers have a positive correlation with the abundance of specialized metabolites in oregano and thyme [17].

**Fig. 9:**
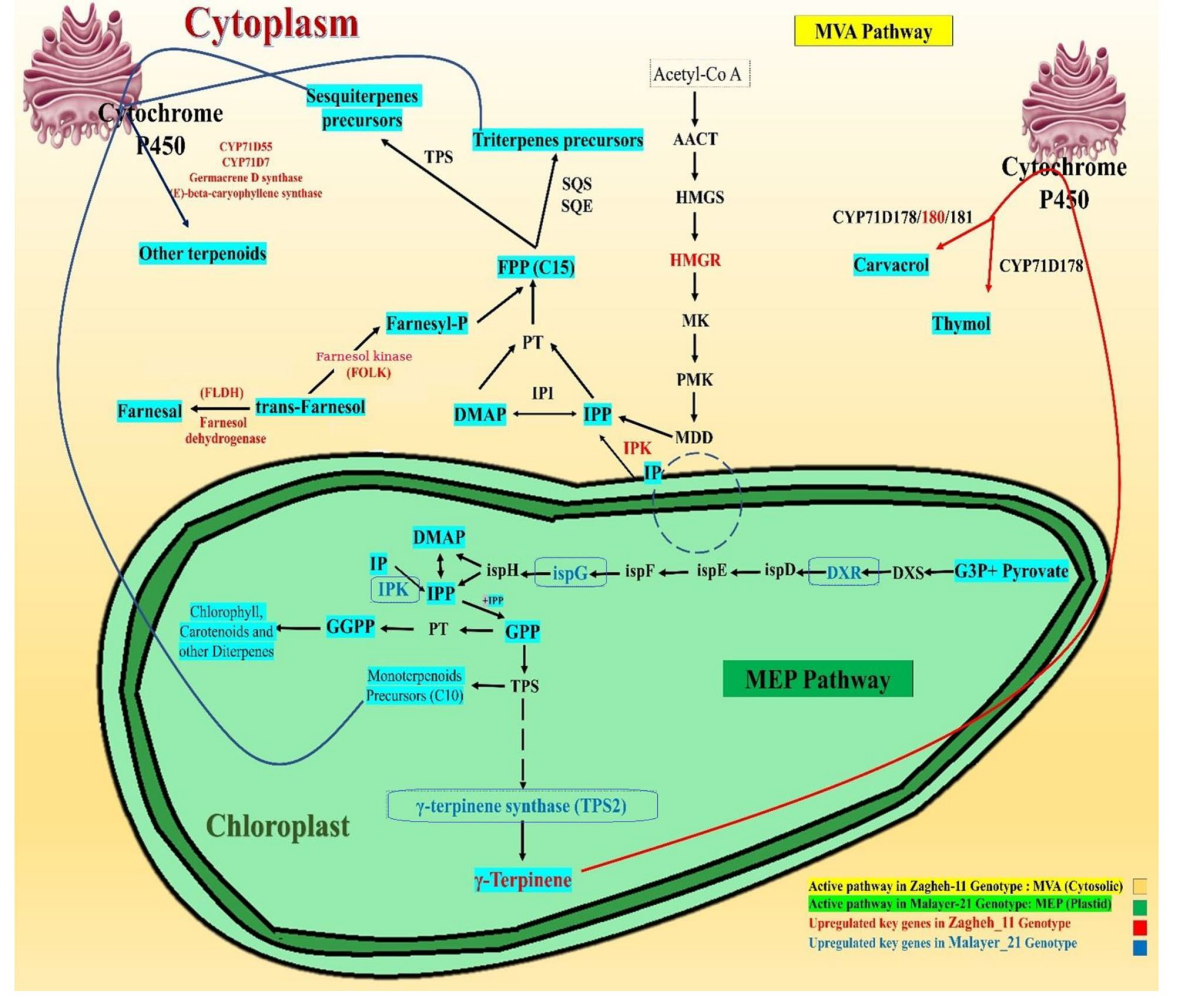
Graphical abstract indicating the active pathways in each clone genotypes (chemotypes) of *T.daenensis*. Blue colored genes were upregulated in Malayer-21 genotype and red colored genes were upregulated in Zagheh-11 genotype. It seems that plastid MEP pathway was more active in Malayer-21, while the cytosolic MVA pathway was boosted in Zagheh-11 genotype

Tracking the biosynthetic trails of bioactive compounds, which may be complex and engaged with several transcriptional regulators and crosstalks, has become a research spotlight in bioprospecting of natural constituents [35]. Development of high-throughput sequencing platforms has provided unprecedented opportunities to explore the reduced representation of genomes and to profile global gene expression patterns in non-model species [31]. In various medicinal plant species, systematic analysis of transcriptomes is being extensively used to comprehend the metabolic pathways, gene functions and their participation in the synthesis of natural products [36]. In particular, screening the entire transcriptomic profile of medicinal species, especially for those with no reference genome, can facilitate discovery of the drivers behind chemical variations [31]. *Thymus* species are exceptional case studies in chemodiversity domains, as over 20 chemotypes have already been described for their EO profiles [37]. To date, several studies have shown that the phytochemical constituents of plant species can be altered due to agronomic practices [38], natural diversity [39], intra- or inter- specific variations [4, 40], abiotic stresses [41], elicitor applications [42], and site-directed mutagenesis in corresponding enzymes [43]. In another study, Vaičiulytė & Ložienė stated that even meteorological factors can significantly influence the amounts of phenolic and non-phenolic monoterpenes in three chemotypes of *Thymus pulegioides* [44]. Despite the extensive literature on the agronomic and phytochemical relevance of *T. daenensis* [1–3], few studies exist on the molecular basis of biosynthesis and variation of secondary metabolites. The transcriptomic data generated herein could shed light on the regulation of some unique transcripts that likely correspond to overactivity of two distinct metabolic pathways. We inferred that the MVA pathway is suitable for the synthesis of more sesquiterpenes, triterpenic acids, and carvacrol. We assumed that the MEP pathway was a major contributor to increased production of thymol-enriched EO. Hypothetically, it has been argued that there is no en-bloc regulation of all genes participating in isoprenoid biosynthesis via the MVA and MEP pathways [20]. Both pathways are independently governed through regulatory networks with limited connectivity and are controlled at transcriptional and protein levels [20]. Although some evidences confirm the crosstalks between these pathways, there is still uncertainty about how much each pathway contributes to synthesizing isoprenoids as the primary skeletons of evolved terpenoids [45]. We found that only a few cooperating genes could induce metabolic differences between the genotypes studied, which was consistent with the statement mentioned above. Regulating all genes at once was not the case, in fact. Only a few key genes, whether in upstream pathways (primary skeletons) or in terminal steps (ultimate steps of synthesizing specialized metabolites), exhibited differential expression patterns (Fig. 9). In an agreement, Qiu et al. [20] stated that the occurrence of five chemotypes in Martaban camphor coincided with differential expression of backbone genes and also different terpene synthases. In another study on *Withania somnifera*, the chemodiversity of whithanolides was driven by the differential expression of genes encoding cytochrome P450s, glycosyltransferase, and methyltransferase enzymes [19].

The synthesis of triterpenic acids (TAs) occurs by cyclizing the linear 30-carbon precursor called squalene, which itself results from the joining of two farnesyl pyrophosphate units [7]. First, *squalene epoxidase* (*SQE*) catalyzes the conversion of squalene to 2,3-oxidosqualene. Specific *oxidosqualene cyclases* (OSCs) then generate a wide array of triterpenoid backbones, including lupeol, α-amyrin, and β-amyrin [7]. These primary carbon skeletons are rearranged by multiple cytochrome P450-dependent monooxygenases (P450s) that introduce a series of hydroxyl, carbonyl, and carboxyl groups to yield the final TAs such as BA, OA, and UA [46]. Based on the results (RNA-seq and qRT-PCR) here, we believe that overexpression of *HMGR*, *FOLK*, and *β-amyrin 28-monooxigenase* genes in ‘Zagheh-11’ may have boosted a metabolic shift towards biosynthesis of heavier triterpenic acids instead of producing volatile monoterpenes. The reduction in EO content in ‘Zagheh-11’ may be due to this change (Fig. S1). In support of our findings, Zeng and Dehesh stated that *HMGR* activity has a considerable effect on metabolic flux of the MVA pathway [47]. They explained that *HMGR* expression in eukaryotic hosts can correlatively enhance accumulation of squalene. On the other hand, *farnesol kinase* (*FOLK*) was also highly expressed in the sesquiterpene/triterpene-rich genotype (‘Zagheh-11’). At higher concentrations, farnesol can be harmful to plant cells. Farnesol is phosphorylated to farnesyl phosphate (FP) and subsequently to FPP [48]. The latter product may thus serve as another potential precursor to increase the amounts of sesquiterpenes and triterpenes in ‘Zagheh-11’ (Fig. 9; Fig. S8). Moreover, *β-amyrin 28-monooxigenase*, which is a multifunctional cytochrome P450-dependent enzyme, was also overexpressed in ‘Zagheh-11’. This enzyme may be responsible for the higher amounts of TAs in ‘Zagheh-11’, as this enzyme catalyzes the carboxylation of *β-*amyrin to α-amyrin and lupeol at the C-28 position to form TAs at the very final steps [46]. Similarly, a unigene encoding another cytochrome P450 enzyme (*CYP71D180*), which catalyzes synthesis of carvacrol from *γ*-terpinene, was abundantly expressed in ‘Zagheh-11’ (a carvacrol-rich genotype) (Fig. 9; Fig. S8). Increased expression of two sesquiterpene syntheses (*germacrene* D and *caryophyllene synthases*) was also observed in ‘Zagheh-11’. Although germacrene D was not observed in gas chromatography analysis, ‘Zagheh-11’ contained greater amounts of caryophyllene and caryophyllene oxide than ‘Malayer-21’. Consistent with these findings, GO enrichment analysis approved that terpene synthase is more activated and represented in ‘Zagheh-11’ than in ‘Malayer-21’ (Fig. 7). Pathway enrichment through KEGG database could unravel considerable differences between these genotypes regarding primary and secondary metabolites (Table 1).

In Malayer-21, we observed high expression of three key genes (*DXR*, *ispG*, and *γ-terpinene synthase*), which was assumed to be a key factor influencing thymol, *γ*-terpinene, and EO content (Fig. 9). ispC (*DXR*), ispG (*HDS*), and *ispH* (*HDR*) are proposed as three important genes that control MEP flux at both initial and terminal steps of isoprenoid synthesis [49–50]. Consistent with the higher amounts of thymol and *γ*-terpinene as a precursor, a higher expression value was found for *γ-terpinene synthase*. Correlation analysis revealed a significant negative relationship (*r* = -0.80, *p* < 0.01) between thymol and *γ*-terpinene content. In addition, a significant positive correlation (*r* = 0.75, *p* < 0.01) was observed between EO and thymol content. These results reinforce the conclusion made by Crocoll et al. [17], who suggested that *γ*-terpinene is the precursor of thymol and carvacrol by examining promiscuity and consumption of substrates in the respective *CYP71D178-182* enzymes.

In the present research, WGCNA analysis facilitated discovery of gene co-expression models and identification of the modules associated with the metabolomes of the genotypes. Blue and brown modules were highly correlated with ‘Zagheh-11’ and its chemical profile, while unigenes found in turquoise and green modules (associated with thymol and EO content) were abundantly expressed in Malayer-21. In metabolic engineering projects, it is worthwhile to review the gene contents of these modules holistically. This information can be employed to elucidate the protein’s interactions with each other and their co-expression behavior.

The expression patterns of 31 TF families were analyzed in the studied samples (Fig. 8). It was observed that the expression of three important TF families (*MYB*, *MYB*-related, and *HD*-*ZIP*) involved in the initiation of glandular secretory trichomes were upregulated in Malayer-21. It can be assumed that the increased density of glandular secretory trichomes, possibly due to the activity of the products of these genes, may have resulted in an enhanced reservoir of EOs in leaves. The *HD*-*ZIP* TFs belong to the homeobox (HB) protein superfamily and consist of homeodomains and an extra leucine zipper domain [51]. The members of this family are involved in numerous biological functions, such as light stress response, polar auxin transduction, cell differentiation, trichome formation, anthocyanin accumulation, meristematic formation, lateral organogenesis, and vascular system development [51]. In genome assembly of *Thymus quinquecostatus*, Sun et al. [52] suggested that initiation of glandular secretory trichomes may be linked to the expression of *MYB* and *HD*-*ZIP* TFs. We also believe that overexpression of *AP2*/*ERF*, *bHLH*, *WRKY*, *MYB*, and *NAC* TF families may be accompanied by higher amounts of thymol and EO in Malayer-21, as confirmed by many studies on specialized metabolites [25–27]. Molecular engineering of these TF genes may be an efficient means to enhance bioproduction of pharmacologically active metabolites [53]. Taken together, our results proposes that six functional genes (*HMGR*, DXR, *CYP71D180*, TPS2, ispG and *β-amyrin 28-monooxygenase*) might be the major drivers of variations in prominent terpenic compounds of *Thymus daenensis* (thymol, carvacrol and triterpenic acids). We highlighted and introduced two distinct metabolic pathways which lead to the emergence of two completely different chemical types as suggested targets for future metabolic engineering. Hopefully, targeted metabolic engineers can recall the enhanced expression of these genes for over-production of these secondary metabolites.

## Conclusion

The current study has established a proper connection between the transcriptome and the terpenome of selected *T. daenensis* genotypes. RNA-seq data could elucidate the expression patterns of certain key genes that play a prominent role in the appearance of two different chemotypes in this species. This study demonstrated how a limited number of gene isoforms can have a major impact on altering metabolic routes and changing the chemical profiles of this plant. High expression levels of some key cytosolic genes, such as *HMGR*, *CYP71D180*, *β-amyrin 28-monooxygenase*, and other *sesquiterpene synthases* may direct metabolism toward biosynthesis of more sesquiterpenes, triterpeneic acids, and carvacrol as a monoterpenic phenols through the MVA pathway. The enhanced expression of some transcription factors (*HD*-*ZIP*, *MYB*, and *MYB* related) may be associated with increased generation of glandular trichomes and a resultant hyperaccumulation of EOs in Malayer-21. Furthermore, the increased levels (even up to approximately log_2_FC > 25) of some chloroplast genes (i.e., *DXR*, *ispG*, and *TdTPD2*) were attributed to the high thymol content in Malayer-21. Although transcriptional regulation may contribute to chemotype appearance, other “omics” approaches (such as genomics, proteomics, or degradomics) have not yet been used to characterize the mechanisms of action driven by these genes. Future functional genomics studies (such as heterologous expression and promoter isolation of the mentioned genes) may benefit from the results generated in this study.

## Materials and methods

### Plant materials and vegetative propagation

Phytochemical and yield characteristics of several *T. daenensis* genotypes (over 150 genotypes) from different ecotypes have been evaluated in the past decade [5, 54]. During the ongoing breeding program, *T. daenensis* genotypes exhibited distinct differences in dry weight, EO content, and phytochemical quantity. Two metabolically extreme genotypes (‘Zagheh-11’ and Malayer-21) were selected from the breeding materials of the medicinal plants core collection based on previous results (Research station of the department of horticultural science, University of Tehran, Karaj, Iran (35°77′ N, 50°92′ E)). 5-cm soft stem cuttings were taken from plant branches to propagate the selected genotypes. Mist spraying (once every 4 h for 1 min) and hormonal application (1000 ppm IBA, Sigma Aldrich, USA) were applied to the cuttings for root emergence in 96-well plastic trays (cocopeat: perlite, 60:40 v:v). At least 20 well-rooted plants with the same genetic identity were present in each of these two clones. The plants were moved to 8-L pots (containing field soil, sand, and leaf mold, 2:1:1 v:v:v) and received the necessary nutritional support in a glass greenhouse. When the plants were well grown, 20 plants of each clone (1 row per clone) were transported and cultivated in the field research station of the department (35°77′ N, 50°92′ E). The distances between rows and plants on a row were 60 and 30 cm, respectively. The plants were irrigated and fertilized using drip strips on a proper schedule by keeping the soil at its own field capacity. To reconfirm the phytochemical divergence of these genotypes, the content of secondary metabolites in aerial parts of the plants were monitored at four different phenological stages (vegetative, bud burst, and early and full-flowering stage). Furthermore, to supply enough plant material for subsequent mRNA-seq and real-time PCR, separate samples from each phenological stage were initially fast-frozen and then stored at -80° until the best phenological stage was determined.

### Essential oil extraction

Initially, aerial parts of three plants (as three biological replications) from each clone at four phenological stages were obtained and dried in an oven at 40 °C. Following the extraction procedure recommended by the British Pharmacopoeia [55], 20 g of dry plant materials were subjected to hydrodistillation using a Clevenger apparatus for 3 h. The redundant water was removed from the EO vials by adding anhydrous sodium sulfate. By calculating the EO volume/dry weight ratio, the EO content (v/w) was obtained. The data related to EO content and constituents were analyzed as a factorial experiment based on completely randomized design (CRD) using SAS software version 9.4 (SAS institute, USA).

### GC-MS and GC-FID analysis of essential oils

Following the optimized method for quantification of EOs in our previous studies [40–41, 57], GC-MS (Agilent 5977 A) and GC-FID (Agilent 7990B) analysis of 24 samples (4 phenological stage × 2 genotype × 3 rep) was performed utilizing an HP-5MS capillary column (5% phenylmethyl polysiloxane, 30 m length, 0.25 mm internal diameter, 0.1 μm film thickness). EO samples were diluted with *n*-hexane (1:100), after which an aliquot of 1 µL was injected. The gradient temperature of the oven in both GC-FID and GC-MS was as follows: 5 min at 60^°^C, subsequently 3^°^C min^− 1^ to reach 150^°^C, and for 1 min stop at 150^°^C. The temperature of the injector and transfer line was determined to be 230^°^C and 240^°^C, respectively. The carrier gas was helium gas with a flow rate of 1 mL min^− 1^. The injector had a split ratio of 1:30, and the MS detector scanned the compound’s mass in the range of 40–400 m/z. To determine EO compositions, a combinational method was performed, including calculation of arithmetic retention indices using coherence of homologous series of hydrocarbons (Supelco, Bellefonte, USA), matching determined retention indices with those given in reference publications [56], and interpretation of mass data with the WILEY 275 and NIST 05 libraries. Moreover, the identity of some constituents was reconfirmed by peak assignment of certified reference standards (thymol, carvacrol, caryophyllene oxide, carvacrol methyl ether, and γ-terpinene, Supleco, USA). The content of each EO compound was expressed as relative percentage of that constituent [57].

### Isolation and HPLC-PDA-assisted quantification of triterpenic acids

To reduce redundant compounds disturbing the quantification procedure of triterpenic acids, a two-step separation was performed as described by Mirjalili et al. [6]. At the early flowering stage, 1 g of powdered dried plant materials was soaked in 40 mL of methanol for 24 h. The methanolic extraction was centrifuged at 5000 rpm for 5 min. Using a rotary evaporator at 40^°^C, the supernatant was isolated, filtered, and finally dried. Then, 15 mL ethyl acetate and 15 mL double-distilled water were added to the dried extraction powder. After concentration through a rotary evaporator, the ethyl acetate fraction was isolated and dried. This dried extract was redissolved in 10 mL HPLC-grade methanol, filtered, and stored at 4 °C until HPLC-PDA analysis. A total of six samples (each genotype 3 replications) was analyzed using a Knauer liquid chromatography system with a C18 analytical column and a 2800 Smart-line photodiode detector (PDA). An isocratic separation method was employed by using methanol:phosphoric acid:water (87:0.05:12.95, v/v/v) as a mobile phase with flow rate 1 mL min^− 1^. Peaks were monitored at 210 nm wavelength [58]. The autosampler injected 20 µL of the extract; all injections were repeated twice. Authentic standards of betulinic acid, oleanolic acid, and ursolic acid (Sigma-Aldrich, USA) were used to plot linear calibration curves (R^2^ = 0.999) by preparing serial dilutions from stock solution at concentration range 10–200 ppm. Three major triterpenic acids were then quantified in the analyzed samples. The final values were calculated in Excel 2020.

### mRNA sequencing, data processing, and *de novo* transcriptome assembly

Based on the phytochemical results, pre-stored pooled samples (two plants) of two genotypes (reflecting the greatest differences with other genotypes) from each clone (genotype) were selected for the RNA sequencing. A total of four samples (harvested at early blossoming stage and from flowering aerial parts of plants) were used for RNA isolation (Qiagen RNeasy plant mini extraction kit, QIAGEN, Hilden, Germany). Briefly, samples with high RNA quality and integrity were used for cDNA library construction (TruSeq standard mRNA preparation kit, Illumina company, CA, USA). The Illumina sequencing machine completed 150-cycle pair-ended sequencing at the end. The data were first inspected using FASTQC v0.11.9 [58] and then trimmed to remove low-quality bases (Q < 30) and adaptor contaminations using Trimmomatic v0.39 [59]. De novo assembly of the dataset was performed by utilizing an ensemble workflow that integrates EvidentialGene pipelines [60] in a concatenation method, as described in our recently published article [31]. The clustered, non-redundant, and final assembled transcripts of the EvidentialGene tool were called unigenes.

### Abundance estimation and differential genes expression (DEG) analysis

The abundance of assembled transcripts (unigenes) was estimated by RSEM [61] software, which first employs Bowtie2 [62] software as an aligner motor to map the reads back to the transcriptome assembly. The RSEM output file included the raw count matrix and normalized count matrix of unigenes. The raw count matrix was analyzed using Bioconductor package DESeq2 [63] in the R Studio environment to identify differentially expressed (DE) unigenes. The raw counts were normalized by the DESeq2 package before DE analysis by using the relative log expression (RLE) method; any expression differences in samples were then discovered. A false discovery rate (FDR) of < 0.05 and a log^2^ fold change value (FC) > 2 were determined to identify DE unigenes. Note that normalized log_2_ FPKM + 1 values related to TFs and DE unigenes involved in terpenoids biosynthesis were used for heatmap plot generation. Weighted gene co-expression network (WGCNA) analysis was also used to uncover the co-expression patterns of DE unigenes and the correlation of gene modules with the analyzed samples using the WGCNA package [64].

### Functional annotation of unigenes and enrichment analysis

The assembled unigenes were used as queries to search against the reference databases. For annotation of unigenes, BLASTx runs were conducted against Kyoto Encyclopedia of Genes and Genomes (KEGG), non-redundant (NR) proteins, SwissProt, TAIR10, EggNog, and Uniref100 protein databases with an e-value cutoff of 1e-5 and one target option. Mapping of the metabolic pathways and functional classification for each unigene was assigned through the KEGG database using KAAS automatic KEGG annotation webservice (https://www.genome.jp/kaas-bin/kaas_main). Gene ontology (GO) classification, enrichment and visualization was conducted through AgriGo (http://systemsbiology.cau.edu.cn/agriGOv2) and REVIGO (http://revigo.irb.hr) online webservers. Enrichment analyses were performed for differentially expressed unigenes. The enriched metabolic pathways among the studied genotypes were determined using the KEGG orthology-based annotation server (KOBAS) (http://kobas.cbi.pku.edu.cn). Using the expression data matrix, a parametric gene enrichment analysis was also performed using Cluster Profiler package in the R studio environment to highlight the enriched GO terms and categories. Moreover, homology searches against Plant TFDB were conducted using the iTAK perl script provided by http://itak.feilab.net to characterize transcription factors.

### Quantitative real-time PCR

An Applied Biosystems® Real-time PCR instrument and 5X HOT FIREPol® EvaGreen® qPCR master mix (Ampliqon, SOLIS BIODYNE, Stonia) were used for quantitative expression analysis of selected unigenes. 5X All-In-One® RT master mix (BioBasic, Canada) was used to synthesize cDNA according to the manufacturer’s instructions. Primers were chosen or redesigned based on transcriptomic data or information reported by Crocoll et al. [17] and Kianersi [65]. Elongation factor 1 (ELF1) alpha was used as a housekeeping gene (Fig. S5). Expression levels (log_2_FC = -ΔΔCt) of unigenes were calculated and visualized by GraphPad Prism (8.4.3) software by using ΔΔCt values.

### Electronic supplementary material

Below is the link to the electronic supplementary material.

Supplementary Material 1

## Data Availability

The datasets generated and analyzed in the current project (PRJNA950567) are deposited in the NCBI SRA repository. The data released publicly are now available at the following accession numbers: (read 1: SRR24037549, read 2: SRR24039021, read 3: SRR24039245, read 4: SRR24040063).

## Abbreviations

FLDH: NADP^+^-dependent farnesol dehydrogenase
FOLK: Farnesol kinase
ispC (DXR): 1-deoxy-D-xylulose 5-phosphate reductoisomerase
ispG (HDS): 2-*C*-methyl-D-erythritol-2,4-cyclodiphosphate reductase
HDS: 1-hydroxy-2-methyl-2-(*E*)-butenyl-4-diphosphate synthase
HDR (ispH): 1-hydroxy-2-methyl-2-(*E*)-butenyl-4-diphosphate reductase
HMGR: 3-Hydroxy-3-methyl-glutaryl-CoA reductase
