# Supplementary material for: RNA-seq analysis reveals narrow differential gene expression in MEP and MVA pathways responsible for phytochemical divergence in extreme genotypes of Thymus daenensis Celak
Source: BMC Genomics. 2024 Mar 4;25:237. doi: 10.1186/s12864-024-10164-x (PMC10913619; doi:10.1186/s12864-024-10164-x)
Supplement: Supplementary file 1 — Supplementary Material 1 [file 12864_2024_10164_MOESM1_ESM.docx]

**Fig S1.** Essential oil content of ‘Malayer-21’ and ‘Zagheh-11’ clones of *T. daenensis* measured at different phenological stages.

**Table S1.** Relative percentage of essential oil components measured at different phenological stages in Malayer-21 and Zagheh-11 clones of *T. daenensis*.

| Zagheh-11 | | | | | | | | Malayer-21 | | | |
| --- | --- | --- | --- | --- | --- | --- | --- | --- | --- | --- | --- |
| N | RI_calculated_ | RI_Adams_ | Compound | Vegetative | Flower bud burst | Early flowering | Full flowering | Vegetative | Flower bud burst | Early flowering | Full flowering |
| 1 | 923 | 924 | *α*-Thujene | 2.53±0.24 | 2.49±0.55 | 1.41±0.19 | 3.51±0.01 | 1.64±0.51 | 1.19±0.24 | 3.6±0.73 | 4.62±1.04 |
| 2 | 930 | 932 | *α*-Pinene | 1.38±0.53 | 1.57±0.51 | 1.22±0.37 | 1.15±0.08 | 0.62±0.13 | 0.58±0.09 | 1.46±0.35 | 1.44±0.36 |
| 3 | 944 | 946 | Camphene | 0.4±0.1 | 0.47±0.29 | 0.24±0.05 | 0.23±0.02 | 0.49±0.06 | 0.57±0.09 | 0.32±0.12 | 0.31±0.15 |
| 4 | 971 | 969 | Sabinene | 0.63±0.2 | 0.39±0.39 | 0.04±0.04 | 0.17±0.06 | 0.35±0.11 | 0.6±0.11 | 0.24±0.03 | 0.28±0.05 |
| 5 | 975 | 974 | *β*-Pinene | 0.49±0.06 | 0.45±1 | 0.31±0.1 | 0.57±0.08 | 0.29±0.07 | 0.36±0.09 | 0.37±0.26 | 0.43±0.1 |
| 6 | 990 | 995 | Myrcene | 0.43±0.31 | 0.2±0.05 | 0.21±0.1 | 0.33±0.11 | 0.72±0.03 | 0.59±0.2 | 0.61± 0.05 | 2.48±1.09 |
| 7 | 1002 | 1003 | *α* -Phellandrene | 0.69±0.22 | 1.13±0.64 | 0.57±0.11 | 0.16±0.04 | 0.4±0.44 | 0.28±0.19 | 0.22±0.03 | 0.16±0.06 |
| 8 | 1009 | 1008 | *Δ*-Carene | 1.16±0.5 | 0.65±0.02 | 0.41±0.14 | 0.49±0.08 | 0.66±0.16 | 0.35±0.02 | 0.71±0.19 | 0.45±0.23 |
| 9 | 1014 | 1015 | *α*-Terpinene | 3.77±0.82 | 3.05±0.06 | 1.62±0.74 | 2.6±0.14 | 1.47±0.36 | 1.12±0.31 | 3.51±0.22 | 3.71±0.52 |
| 10 | 1020 | 1021 | ***p*-Cymene** | **8.78±1.17** | **6.1±0.93** | **2.74±0.75** | **7.03±0.47** | **6±1.19** | **3.78±0.62** | **2.76±0.35** | **2.65±0.33** |
| 11 | 1027 | 1026 | 1,8-Cineole | 0.77±0.16 | 0.49±0.32 | 0.23±0.14 | 1.03±0.07 | 0.72±0.14 | 0.53±0.04 | 1.08±0.16 | 0.58±0.16 |
| 12 | 1045 | 1044 | *β*-Ocimene | 0.52±0.15 | 0.39±0.06 | 0.1±0.07 | 0.18±0.07 | 0.32±0.07 | 0.07±0.03 | 0.2±0.06 | 0.23±0.04 |
| 13 | 1054 | 1054 | **γ-Terpinene** | **21.07±1.8** | **17.42±0.57** | **6.19±0.58** | **9.86±1.4** | **15.7±0.55** | **11.19±0.48** | **9.19±0.41** | **8.83±0.44** |
| 14 | 1063 | 1065 | Cis-Sabinene hydrate | 0.52±0.19 | 0.62±0.07 | 0.33±0.16 | 0.83±0.03 | 0.38±0.09 | 0.32±0.16 | 1.34±0.45 | 1.38±0.25 |
| 15 | 1087 | 1086 | *α*-Terpinolene | 0.38±0.23 | 0.39±0.25 | 0.25±0.09 | 0.15±0.02 | 0.3±0.12 | 0.33±0.1 | 0.2± 0.01 | 0.26±0.06 |
| 16 | 1096 | 1096 | Linalool | 0.93±0.47 | 0.34±0.28 | 0.2±0.07 | 0.43±0.09 | 0.36±0.12 | 0.26±0.09 | 0.49±0.09 | 0.41±0.03 |
| 17 | 1097 | 1098 | *trans*-Sabinene hydrate | 0.46±0.16 | 0.23±0.11 | 0.29±0.06 | 0.57±0.06 | 0.43±0.19 | 0.27±0.04 | 0.49±0.19 | 0.52±0.17 |
| 18 | 1240 | 1241 | Carvacrol methyl ether | 3.25±0.82 | 2.76±0.76 | 1.81±0.26 | 1.00±0.65 | 3.72±0.41 | 3.04±0.4 | 4.77±0.39 | 5.36±0.94 |
| 19 | 1288 | 1289 | **Thymol** | **38.98±1.37** | **46.29±3.56** | **52.39±1.83** | **50.79±1.24** | **58.35±1.53** | **67.98±1.56** | **62.16±1.67** | **62.01±2.97** |
| 20 | 1297 | 1298 | **Carvacrol** | **6.6±0.39** | **10.07±1.41** | **26.14±1.98** | **14.37±1.39** | **1.43±0.11** | **1.41±1.15** | **1.12±0.36** | **0.55±0.61** |
| 21 | 1415 | 1417 | (E)-*β*-Caryophyllene | 1.03±0.03 | 1.16±0.01 | 1.43±0.09 | 1.11±0.09 | 1.07±0.02 | 1.07±0.05 | 1.15±0.04 | 1.15±0.06 |
| 22 | 1448 | 1452 | *α-*Humulene | 0.18±0.02 | 0.21±0.04 | 0.3±0.01 | 0.27±0.03 | 0.1±001 | 0.12±0.02 | 0.16±0.02 | 0.18±0.02 |
| 23 | 1504 | 1506 | *α-*Bisabolene | 0.55±0.08 | 0.66±0.14 | 0.85±0.01 | 0.71±0.07 | 0.3±0.04 | 0.37±0.08 | 0.5±0.07 | 0.56±0.06 |
| 24 | 1579 | 1582 | Caryophyllene oxide | 0.2±0.04 | 0.25±0.07 | 0.35±0.04 | 0.26±0.02 | 0.07±0.02 | 0.1±0.04 | 0.17±0.03 | 0.2±0.03 |
|  | | | Total | 95.64 | 97.77 | 99.59 | 97.82 | 95.89 | 96.46 | 96.71 | 98.75 |

**Fig S2.** Gene ontolgy functional classification of assembled unigenes. Each bar represent the number of assigned unigenes to each GO terms.


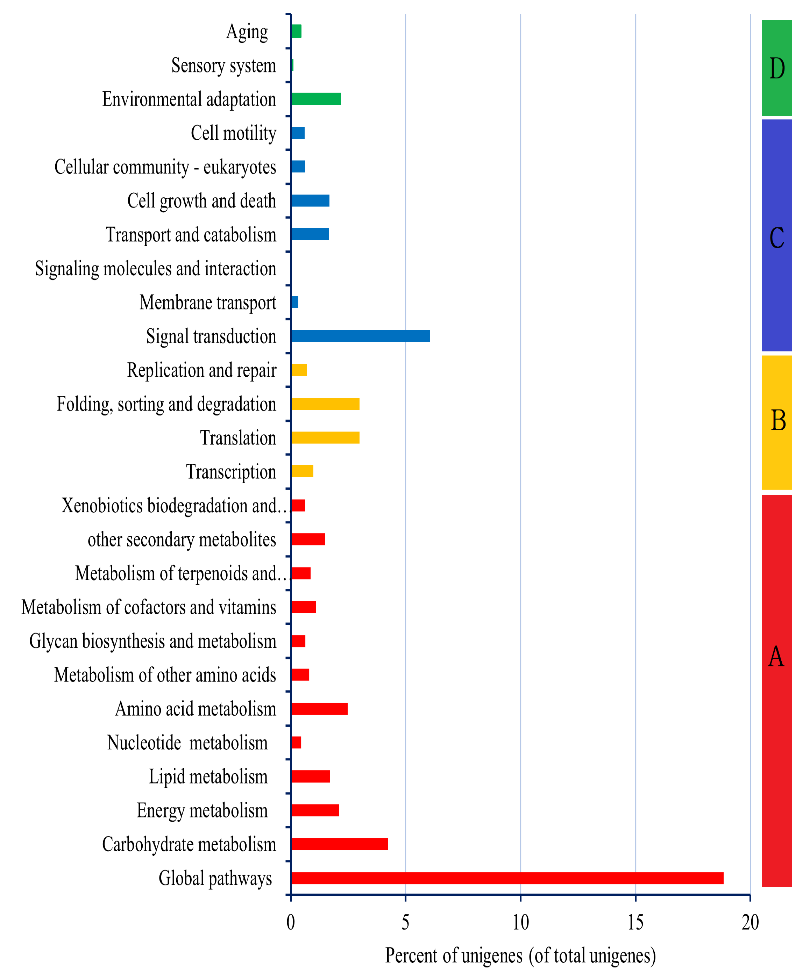


**Fig. S3.** Functional classification of KEGG terms assigned for assembled unigenes. Subcategories are summarized in four main domains: A, Metabolism; B, Genetic Information Processing; C, Cellular Processes; E, Organismal Systems. The y-axis contains the name of the KEGG pathways. The x-axis indicates the percentage of genes annotated under that pathway to the total number of annotated unigenes.

**Table S2.** Information on global and main KEGG pathway maps assigned for assembled unigenes.

| Pathways | References KEGG map ID (KO) | No. of genes in each pathway | No. of identified genes in each pathway | Percent of identified genes in each pathway | No. of identified unigenes (orthologs) in each KEGG pathway |
| --- | --- | --- | --- | --- | --- |
| Metabolic pathways | 110 | 2260 | 1007 | 45 | 25853 |
| Biosynthesis of secondary metabolites | 1110 | 995 | 506 | 51 | 15234 |
| Carbon metabolism | 1200 | 342 | 98 | 29 | 3895 |
| 2-Oxocarboxylic acid metabolism | 1210 | 75 | 30 | 40 | 812 |
| Fatty acid metabolism | 1212 | 70 | 27 | 39 | 875 |
| Biosynthesis of amino acids | 1230 | 227 | 99 | 44 | 3090 |

| Category | Pathways | Referencemap ID (KO) | N. of genes in each KEGG pathway | N. of identified genes in each KEGG pathways | Percent of identified genes in each pathway | N. of unigenes (orthologs) assigned for each pathway |  |
| --- | --- | --- | --- | --- | --- | --- | --- |
| Metabolism of terpenoids and polyketides | | Terpenoid backbone biosynthesis | 900 | 53 | 31 | 58 | 776 |
|  |  | Monoterpenoid biosynthesis | 902 | 23 | 6 | 26 | 289 |
|  |  | Sesquiterpenoid and triterpenoid biosynthesis | 909 | 66 | 11 | 17 | 306 |
|  |  | Diterpenoid biosynthesis | 904 | 42 | 10 | 24 | 239 |
|  |  | Carotenoid biosynthesis | 906 | 46 | 20 | 43 | 347 |
|  |  | Brassinosteroid biosynthesis | 905 | 10 | 9 | 9 | 139 |
| Biosynthesis of some other important secondary metabolites | | Phenylpropanoid biosynthesis | 940 | 33 | 16 | 45 | 1512 |
|  |  | Stilbenoid, and gingerol biosynthesis | 945 | 13 | 5 | 38 | 532 |
|  |  | Flavonoid biosynthesis | 941 | 19 | 14 | 74 | 714 |
|  |  | Flavone and flavonol biosynthesis | 944 | 12 | 5 | 42 | 95 |
|  |  | Anthocyanin biosynthesis | 942 | 14 | 3 | 21 | 43 |
|  |  | Isoflavonoid biosynthesis | 943 | 13 | 5 | 38 | 100 |
|  |  | Indole alkaloid biosynthesis | 901 | 10 | 2 | 20 | 39 |
|  |  | Isoquinoline alkaloid biosynthesis | 950 | 42 | 11 | 26 | 438 |
|  |  | Tropane, piperidine and pyridine alkaloids | 960 | 26 | 11 | 42 | 370 |

**Table S3.** KEGG annotation for assembled unigenes related to the secondary metabolite pathways.

| Terpenoid backbone biosynthesis | Identified genes in metabolic map |
| --- | --- |
| ko:K00021 | HMGR; hydroxymethylglutaryl-CoA reductase (NADPH) [EC:1.1.1.34] |
| ko:K00099 | DXR; 1-deoxy-D-xylulose-5-phosphate reductoisomerase [EC:1.1.1.267] |
| ko:K00587 | ICMT; protein-S-isoprenylcysteine O-methyltransferase [EC:2.1.1.100] |
| ko:K00626 | ACAT; acetyl-CoA C-acetyltransferase [EC:2.3.1.9] |
| ko:K00787 | FDPS; farnesyl diphosphate synthase [EC:2.5.1.1 2.5.1.10] |
| ko:K00869 | MVK; mevalonate kinase [EC:2.7.1.36] |
| ko:K00919 | ispE; 4-diphosphocytidyl-2-C-methyl-D-erythritol kinase [EC:2.7.1.148] |
| ko:K00938 | E2.7.4.2; phosphomevalonate kinase [EC:2.7.4.2] |
| ko:K00991 | ispD; 2-C-methyl-D-erythritol 4-phosphate cytidylyltransferase [EC:2.7.7.60] |
| ko:K01597 | MVD; diphosphomevalonate decarboxylase [EC:4.1.1.33] |
| ko:K01641 | HMGCS; hydroxymethylglutaryl-CoA synthase [EC:2.3.3.10] |
| ko:K01662 | DXS; 1-deoxy-D-xylulose-5-phosphate synthase [EC:2.2.1.7] |
| ko:K01770 | ispF; 2-C-methyl-D-erythritol 2,4-cyclodiphosphate synthase [EC:4.6.1.12] |
| ko:K01823 | idi; isopentenyl-diphosphate Delta-isomerase [EC:5.3.3.2] |
| ko:K03526 | gcpE; (E)-4-hydroxy-3-methylbut-2-enyl-diphosphate synthase [EC:1.17.7.1 1.17.7.3] |
| ko:K03527 | ispH; 4-hydroxy-3-methylbut-2-en-1-yl diphosphate reductase [EC:1.17.7.4] |
| ko:K05356 | SPS; all-trans-nonaprenyl-diphosphate synthase [EC:2.5.1.84 2.5.1.85] |
| ko:K05906 | PCYOX1; prenylcysteine oxidase / farnesylcysteine lyase [EC:1.8.3.5 1.8.3.6] |
| ko:K05954 | FNTB; protein farnesyltransferase subunit beta [EC:2.5.1.58] |
| ko:K05955 | FNTA; protein farnesyltransferase/geranylgeranyltransferase type-1 subunit alpha [EC:2.5.1.58 2.5.1.59] |
| ko:K06013 | STE24; STE24 endopeptidase [EC:3.4.24.84] |
| ko:K06981 | ipk; isopentenyl phosphate kinase [EC:2.7.4.26] |
| ko:K08658 | RCE1; prenyl protein peptidase [EC:3.4.22.-] |
| ko:K10960 | chlP; geranylgeranyl diphosphate reductase [EC:1.3.1.83 1.3.1.111] |
| ko:K11778 | DHDDS; ditrans,polycis-polyprenyl diphosphate synthase [EC:2.5.1.87] |
| ko:K12742 | ispS; isoprene synthase [EC:4.2.3.27] |
| ko:K13789 | GGPS; geranylgeranyl diphosphate synthase, type II [EC:2.5.1.1 2.5.1.10 2.5.1.29] |
| ko:K14066 | GPS; geranyl diphosphate synthase [EC:2.5.1.1] |
| ko:K15889 | PCME; prenylcysteine alpha-carboxyl methylesterase [EC:3.1.1.-] |
| ko:K15891 | FLDH; NAD+-dependent farnesol dehydrogenase [EC:1.1.1.354] |
| ko:K15892 | FOLK; farnesol kinase [EC:2.7.1.216] |
| Monoterpens and diterpenoids (except gibberellin) | Identified genes in metabolic map |
| ko:K07385 | TPS-Cin; 1,8-cineole synthase [EC:4.2.3.108] |
| ko:K15095 | E1.1.1.208; (+)-neomenthol dehydrogenase [EC:1.1.1.208] |
| ko:K18108 | E4.2.3.111; (-)-alpha-terpineol synthase [EC:4.2.3.111] |
| ko:K21925 | TPS3; camphene/tricyclene/(4S)-limonene/myrcene synthase [EC:4.2.3.117 4.2.3.105 4.2.3.16 4.2.3.15] |
| ko:K23232 | 10HGO; 8-hydroxygeraniol dehydrogenase [EC:1.1.1.324] |
| ko:K23810 | CYP76F14; (E)-8-carboxylinalool synthase [EC:1.14.14.-] |
| ko:K17982 | TPS04; geranyl-linalool synthase [EC:4.2.3.144] |
| Sesquiterpenoid and triterpenoid | Identified genes in metabolic map |
| ko:K00511 | SQLE; squalene monooxygenase [EC:1.14.14.17] |
| ko:K00801 | FDFT1; farnesyl-diphosphate farnesyltransferase [EC:2.5.1.21] |
| ko:K14173 | AFS1; alpha-farnesene synthase [EC:4.2.3.46] |
| ko:K14175 | NES1; (3S,6E)-nerolidol synthase [EC:4.2.3.48] |
| ko:K14182 | HVS; vetispiradiene synthase [EC:4.2.3.21] |
| ko:K15472 | CYP71D55; premnaspirodiene oxygenase [EC:1.14.14.151] |
| ko:K15803 | GERD; (-)-germacrene D synthase [EC:4.2.3.75] |
| ko:K15805 | EAH; 5-epiaristolochene 1,3-dihydroxylase [EC:1.14.14.149] |
| ko:K15813 | LUP4; beta-amyrin synthase [EC:5.4.99.39] |
| ko:K15891 | FLDH; NAD+-dependent farnesol dehydrogenase [EC:1.1.1.354] |
| ko:K20659 | LUS; lupeol synthase [EC:5.4.99.41] |

**Table S4.** List of identified genes in biosynthesis pathways of terpenoids (KEGG maps), their KO and enzyme EC number.


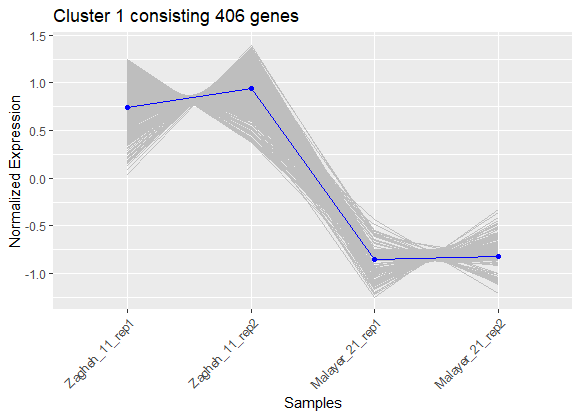

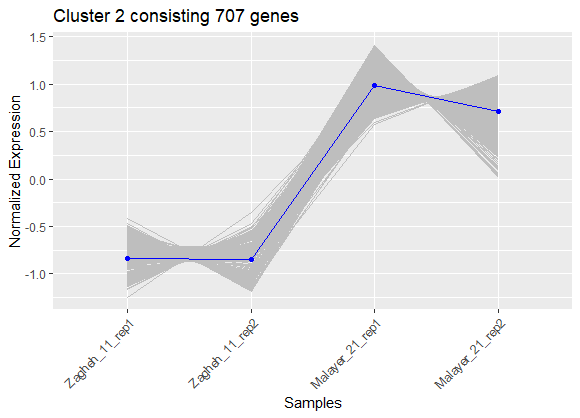

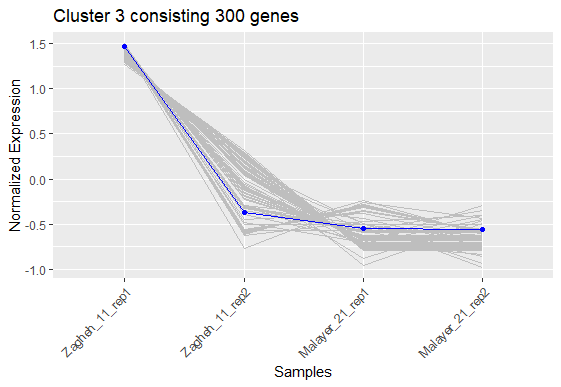

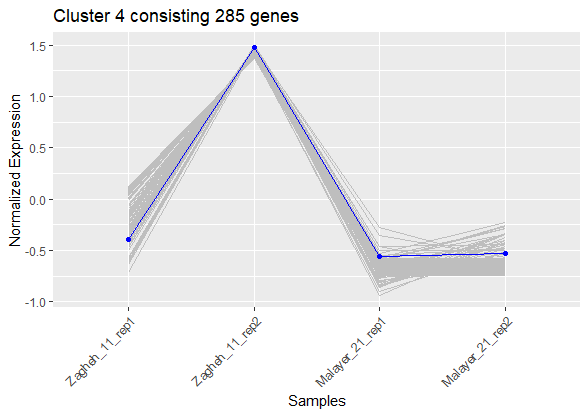

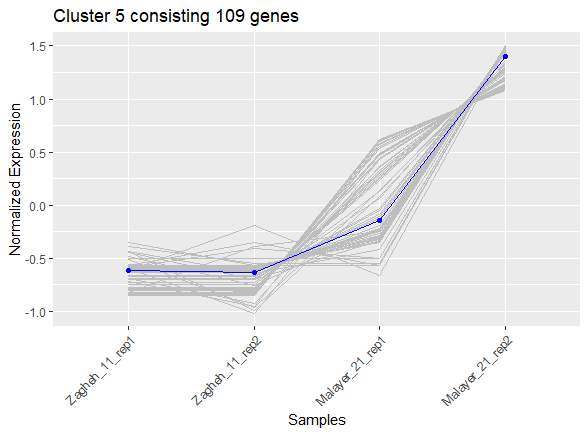

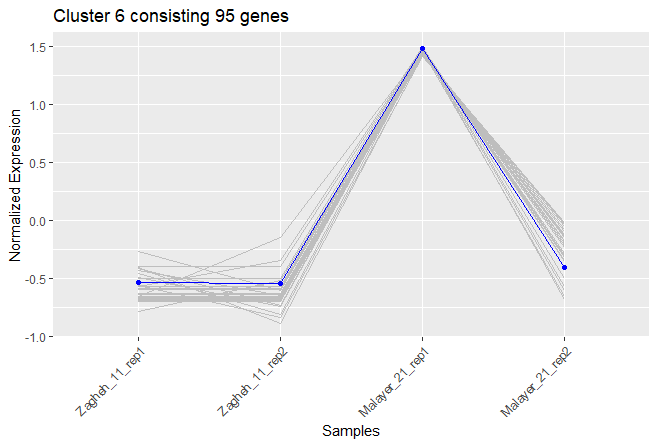


**Fig S4.** Normalized expression patterns of six clusters related to the unigenes showing similar expression patterns in analyzed samples. Normalization of gene expression values were based on variance stabilization.


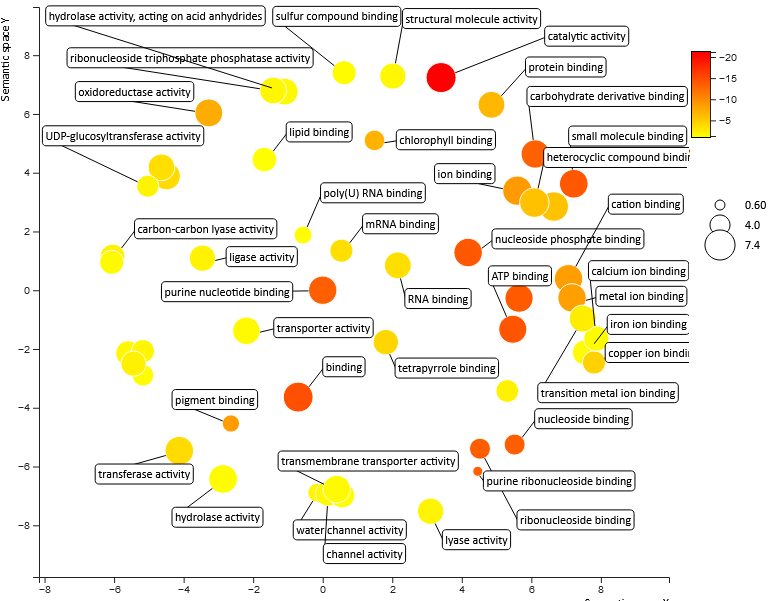

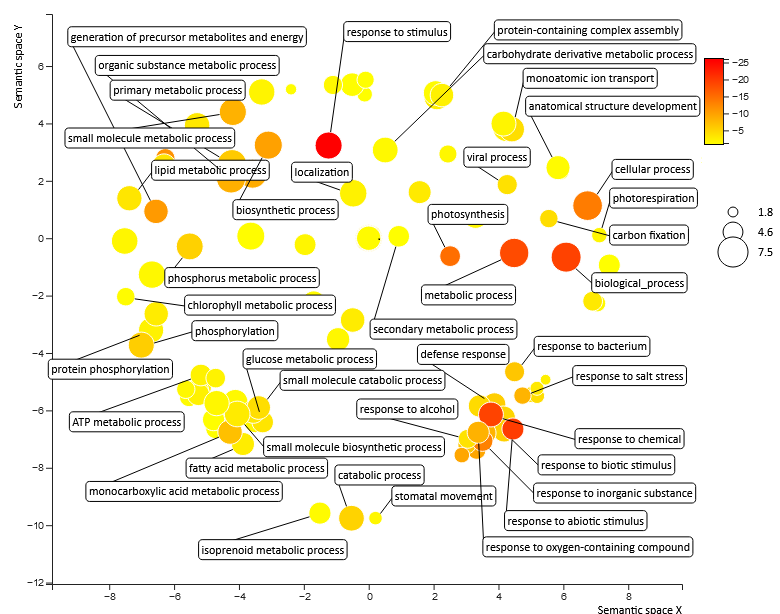


**Fig S5.** GO category enrichment analysis of differentially expressed unigenes related to molecular functions (left) and biological processes (right) in Zagheh-11 vs Malayer-21 comparison**.** Circles depicted by filled color show significantly enriched GO terms with log_10_ p-value <0.05. The colour bubbles represent log_10_ p-value for the GO terms in the underlying databases run through REVIGO webserver (larger size bubbles present having more related unigenes ).


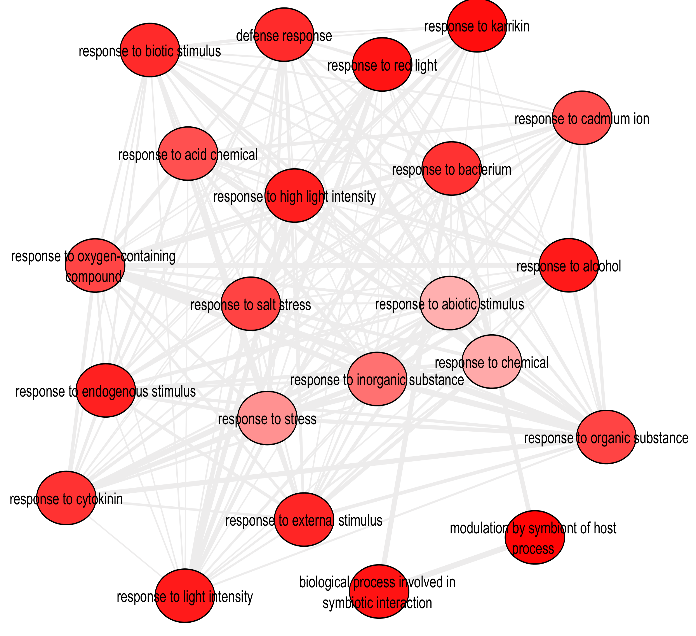

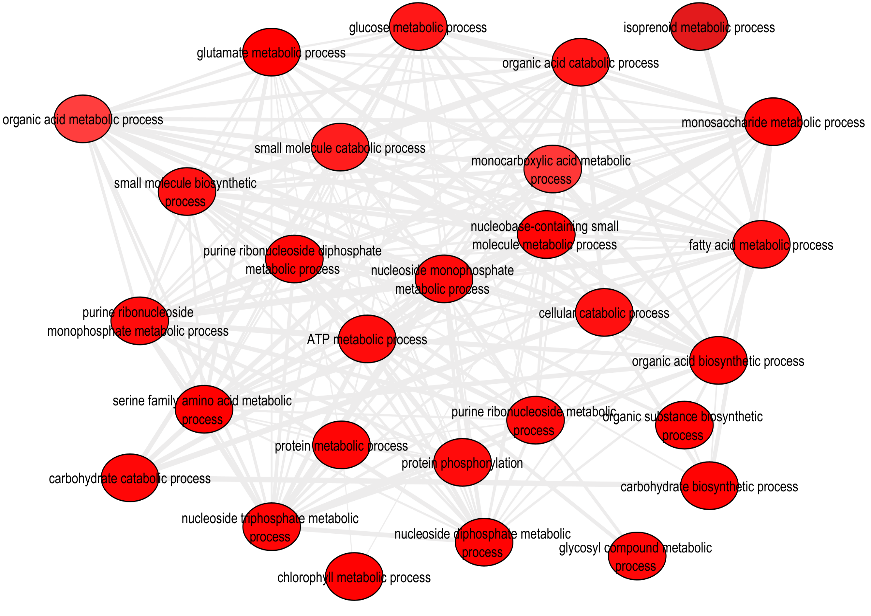


**Figure S6.** The interaction of GO terms in enrichment analysis of differentially expressed genes related to the biological process (BP). This figure was produced by REVIGO and adjusted by Cytoscape. The colour of bubbles shows the p-value of the GO term in related database. Highly similar GO terms are linked together.

**Figure S7.** The number of identified members for transcription factor families in the assembled transcriptome of *T. daenensis*.


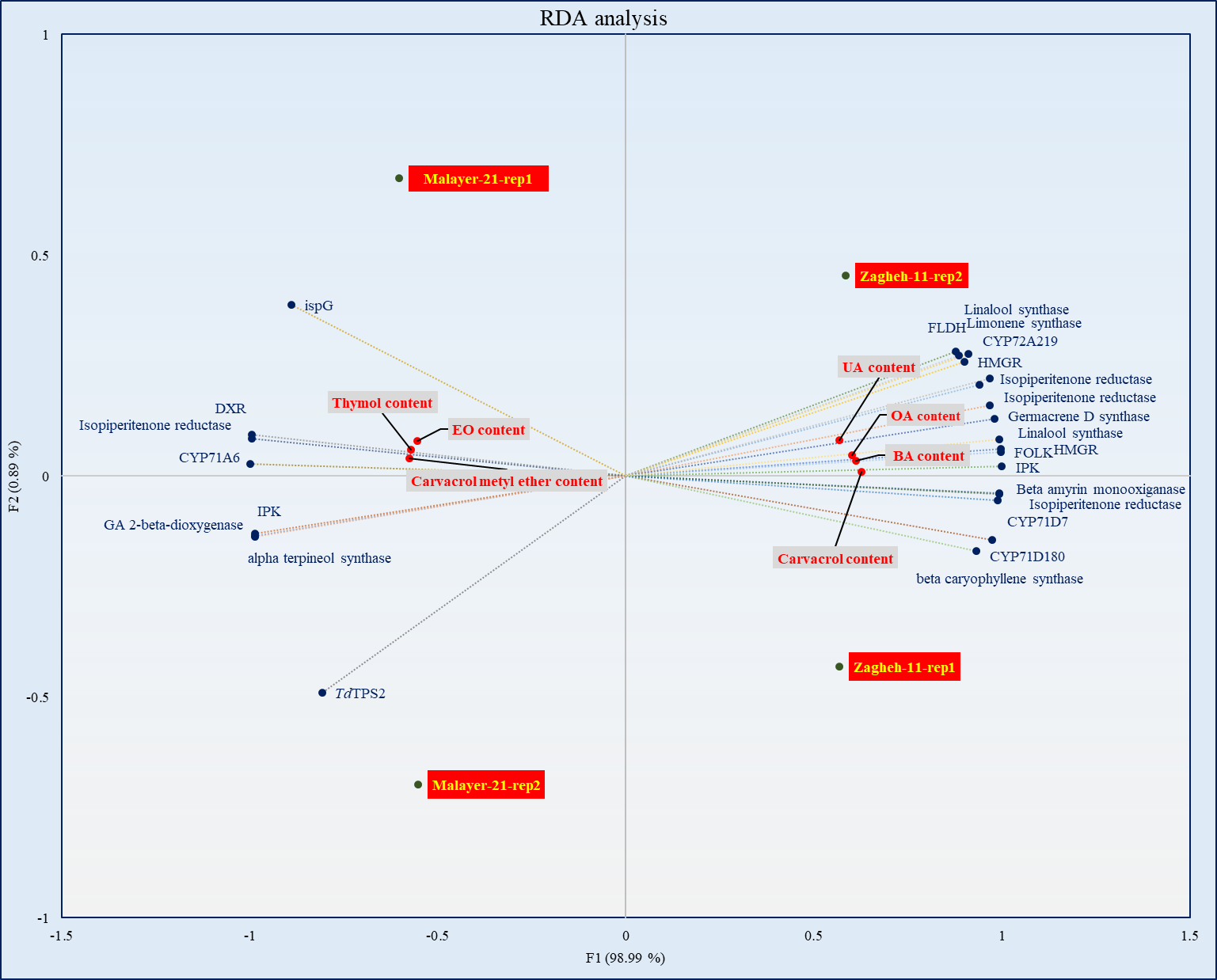


**Fig S8.** Redundancy analysis (RDA) of secondary metabolite content and their related genes.

**Table S5.** The characteristics of isoform-specific primers designed for utilizing in qRT-PCR analysis.

| Real time PCR primers | Direction | Sequence | Tm (°C) | Primer length | Amplicon size (bp) |
| --- | --- | --- | --- | --- | --- |
| CYP71D180 (Evigene261541) | Forward  Reverse | GCAAAGAAGAATGCGAGGTC  GATTGAACGTGTCGGGATCT | 58.4  58.4 | 20  19 | 116 |
| *Td*TPS2 (Evigene94215) | Forward  Reverse | AACCTCGCCGAGAAACTCCC  AGCTGCAGTTCGTCGAGTGT | 62.5  60.5 | 20  20 | 182 |
| HMGR (Evigene1924, Evigene74151) | Forward  Reverse | TTGGTTTGGGTAGTGAGCATC  GCATCTGCTTCTTTCCTTCCT | 59.4  59.4 | 21  21 | 154 |
| DXR (Evigene12571) | Forward  Reverse | AGAGGCTCACTATTTGTTTGGG  CACTGATGAATCCTGTGTTTCG | 60.3  60.3 | 22  22 | 104 |
| ELF-1 (gene-specific, reference gene) | Forward  Reverse | CTCCAGTTCTTGATTGCCACAC  GGTGGGTACTCGGAGAATGTC | 62.1  63.3 | 22  21 | 187 |
